# High-throughput estimation of allele frequencies using combined pooled-population sequencing and haplotype-based data processing

**DOI:** 10.1186/s13007-022-00852-8

**Published:** 2022-03-21

**Authors:** Michael Schneider, Asis Shrestha, Agim Ballvora, Jens Léon

**Affiliations:** 1 Institute of Crop Science and Resource Conservation, University of Bonn, Plant Breeding, Katzenburgweg 5, 53115 Bonn, Germany; 2 Present Address: Institute for Quantitative Genetics and Genomics of Plants, University Duesseldorf, Universitätsstraße 1, 40225 Düsseldorf, Germany

**Keywords:** Pool sequencing, Genotyping, Allele frequency estimation, Single nucleotide polymorphisms, Haplotype, *Hordeum vulgare*

## Abstract

**Background:**

In addition to heterogeneity and artificial selection, natural selection is one of the forces used to combat climate change and improve agrobiodiversity in evolutionary plant breeding. Accurate identification of the specific genomic effects of natural selection will likely accelerate transfer between populations. Thus, insights into changes in allele frequency, adequate population size, gene flow and drift are essential. However, observing such effects often involves a trade-off between costs and resolution when a large sample of genotypes for many loci is analysed. Pool genotyping approaches achieve high resolution and precision in estimating allele frequency when sequence coverage is high. Nevertheless, high-coverage pool sequencing of large genomes is expensive.

**Results:**

Three pool samples (n = 300, 300, 288) from a barley backcross population were generated to assess the population's allele frequency. The tested population (BC_2_F_21_) has undergone 18 generations of natural adaption to conventional farming practice. The accuracies of estimated pool-based allele frequencies and genome coverage yields were compared using three next-generation sequencing genotyping methods. To achieve accurate allele frequency estimates with low sequence coverage, we employed a haplotyping approach. Low coverage allele frequencies of closely located single polymorphisms were aggregated into a single haplotype allele frequency, yielding 2-to-271-times higher depth and increased precision. When we combined different haplotyping tactics, we found that gene and chip marker-based haplotype analyses performed equivalently or better compared with simple contig haplotype windows. Comparing multiple pool samples and referencing against an individual sequencing approach revealed that whole-genome pool re-sequencing (WGS) achieved the highest correlation with individual genotyping (≥ 0.97). In contrast, transcriptome-based genotyping (MACE) and genotyping by sequencing (GBS) pool replicates were significantly associated with higher error rates and lower correlations, but are still valuable to detect large allele frequency variations.

**Conclusions:**

The proposed strategy identified the allele frequency of populations with high accuracy at low cost. This is particularly relevant to evolutionary plant breeding of crops with very large genomes, such as barley. Whole-genome low coverage re-sequencing at 0.03 × coverage per genotype accurately estimated the allele frequency when a loci-based haplotyping approach was applied. The implementation of annotated haplotypes capitalises on the biological background and statistical robustness.

**Supplementary Information:**

The online version contains supplementary material available at 10.1186/s13007-022-00852-8.

## Background

Next-generation sequencing (NGS) technology is routinely used in plant research to detect nucleotide polymorphisms between genotypes at genomic and transcriptomic levels [1–3]. NGS can be applied for various tasks and has been promoted to be of tremendous use in pooled experimental designs. Especially when the number of samples to sequence becomes very high, pooling approaches can lead to a cost reduction of several magnitudes. Pooling strategies have shown high reliability in the detection of rare variants [4] and SNP calling for multiple individual testing [5] and, furthermore were proposed to present an opportunity for genetic map estimation [6]. Population studies, breeding process observation, evolution and adaptation detection, and allele mining approaches can capitalise from the application of pooled sequencing [7, 8]. Population pool sequencing approaches were already applied for small to medium-sized genomes to identify gene-level variations between populations or treatments [9–13]. Studies published in the past couple of years validated different pool sequencing methods, including genotyping by sequencing [7, 8], transcriptome sequencing [14] and whole-genome re-sequencing [15]. The pooled sample sizes were rather small, and the sequencing coverage of the pools was relatively high for all these approaches. For crop species like barley or wheat, having a genome size of approximately 5 GB [16] ⁠and 16 GB [17], respectively, doubling sequencing depth results in severe cost inflation.

However, pooled sequencing has undeniable disadvantages, as the information of haplotypes and heterozygosity is lost. Some approaches try to recover this information [18–20], where the haplotype information of single genotypes has to be derived, and reads need to be rather long to estimate the haplotypes correctly. On top, the identification of rare alleles might be challenging [21]. In addition, the workflow of pooling approaches is more prone to errors compared to single genotype sequencing. The first is the potential unequal contribution of individuals to the pool. Non-uniformity of the individual contribution of DNA or RNA leads to a biased minor allele frequency (MAF) estimation [22]. Tissue and genomic material pooling were reported to produce equivalently uniform pools. Therefore the point of pooling can be chosen concerning cost and labour reduction [18]. Another error can occur from the sequencing depth, making it difficult to estimate the actual allele frequency, especially when an allele's frequency is low. Recommendations on the minimum sequencing coverage range from 50 to 100 reads per polymorphism [23].

In contrast to a static coverage level, Rellstab et al. [21] proposed a minimum coverage adapted to the number of individuals in a pool and the ploidy level. Another approach assesses the actual allele frequency on low coverage levels with only 5 × per locus [24]. The third potential error derives from the sequencing, especially by the amplification step. PCR duplicates can cause a substantial bias in the frequency estimation. This makes it necessary to remove duplicates from the data, which is not always possible [25]. The last important source of error can be the pool size itself. Smaller pools with fewer individuals tend to have a more considerable variation of the individual contribution than bigger pools [18, 26]. The accuracy of pooling approaches has been reported to increase marginally with a sample size exceeding 200 genotypes per pool [25]. However, sequencing techniques as a potential source of errors have not been reported. A further requirement to give a decent overview over a population is a high number of tracked polymorphisms, uniformly distributed over the whole genome with a delicate coverage. Furthermore, the number of individuals pooled should be high to cover the population variation as precisely as possible.

Our goal was to make a reliable allele frequency estimate in *Hordeum vulgare* L*.,* a crop species with a large genome. This study evaluates three next-generation sequencing-based genotyping techniques on a low-level coverage to distinguish the most reliable method with the best costs and coverage trade-offs. Furthermore, four haplotyping approaches were established and compared to precisely estimate the allele frequency of genes or small genomic regions on a low coverage sequencing level.

## Results

**Fig. 1:**
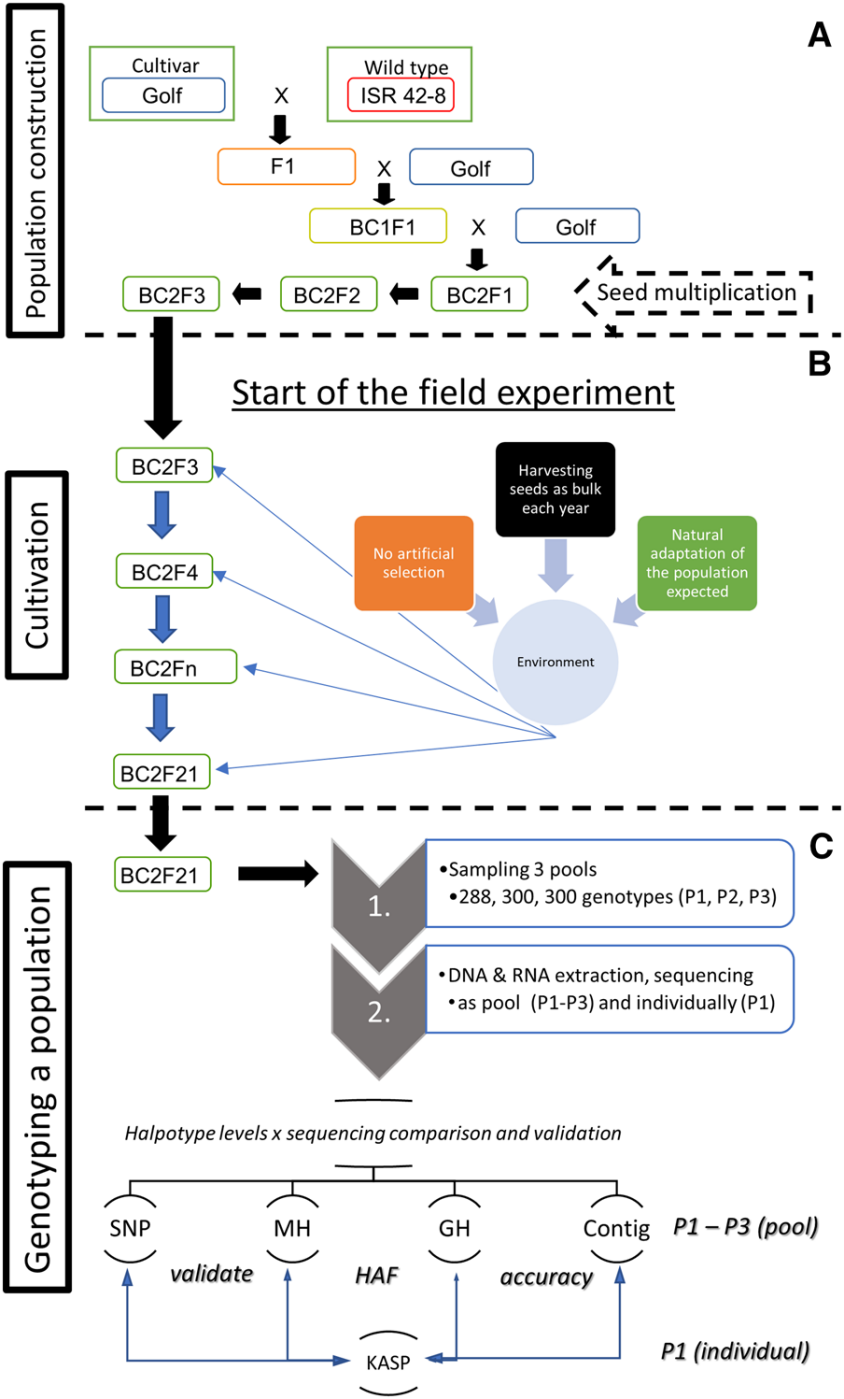
workflow. **A** crossing and establishment of the spring barley population. **B** the field experiment with environmental factors, treatment, and generations. **C** Population genotyping procedure and comparison of different methods

**Fig. 2:**
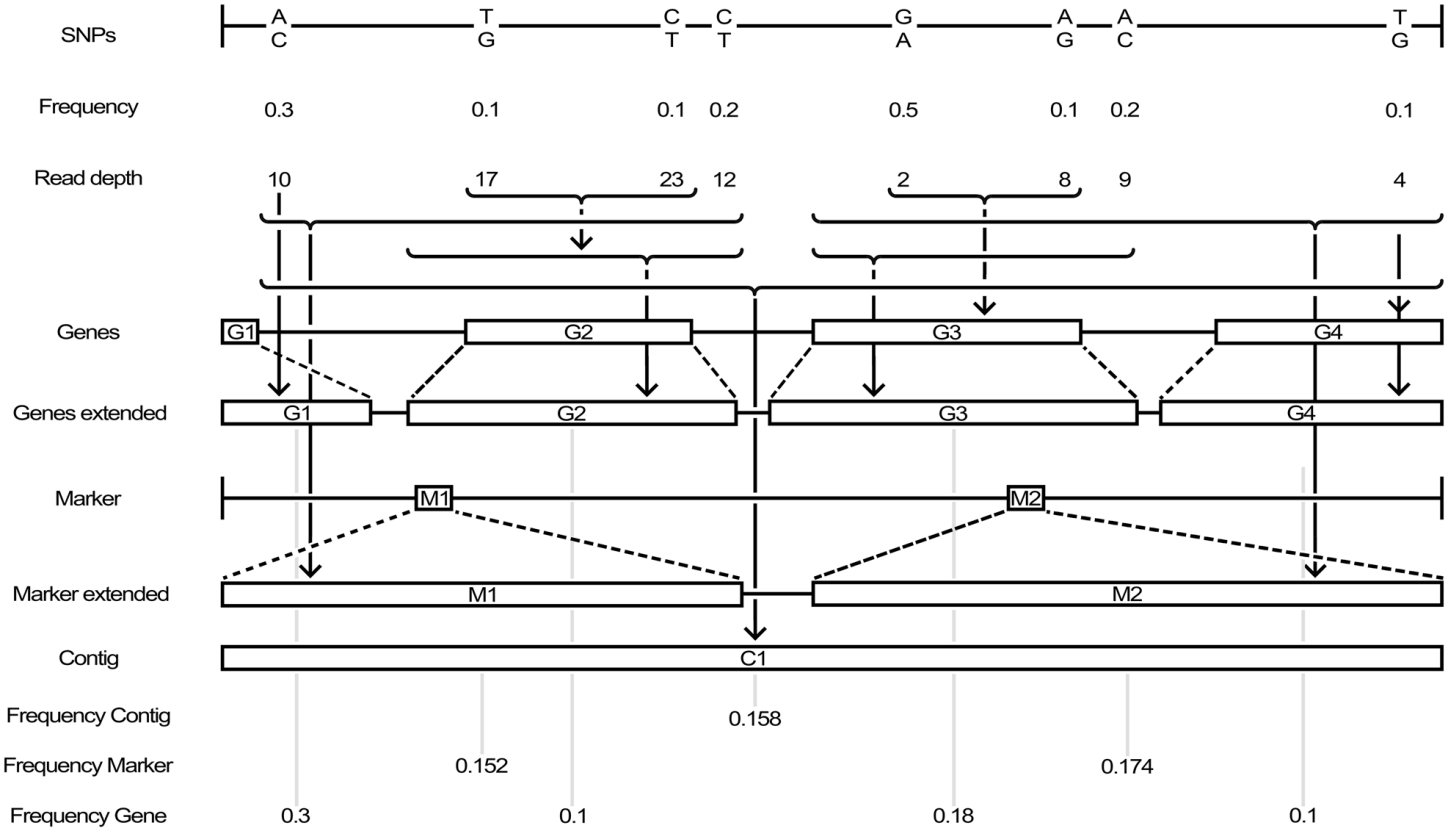
Gene extension algorithm scheme of gene- and marker-based haplotype calculations. The raw SNP-based allele frequency (2nd row; “Raw Frequency”) for identified SNPs (1st row) are identified at a given read depth (3rd row; “Read depth”). Annotated genes (4th row; “Genes”) and markers (6th row; “Markers”) are extended in size up- and downstream (“Genes extended”; “Marker extended”) to associate SNPs in the particular region to the genes / markers. By the extension, more SNPs can be annotated to a gene/marker than without (dashed lines and arrows below the 3rd row indicate relationships). Reported frequency in the last two rows of markers and genes are the calculated haplotype frequency for the wild donor parent in the population. The marker and gene-based haplotype calculation is illustrated in the methods section. The figure should only illustrate the model of SNP information aggregation and does not contain real data

A BC_2_F_21_ barley population, grown in conventional agriculture for eighteen generations, was used to test the hypothesis of accurate allele frequency estimation for an entire population (Fig. 1). The BC_2_F_3_ was the first generation growing in the field after two rounds of unbiased seed multiplication in a greenhouse. No intended artificial selection was performed, and the plots were harvested as bulk to establish next year’s generations. Three pool samples of leaf tissue were created (P1–n = 288; P2–n = 300, P3–n = 300), DNA and RNA extracted and genotyped by three low coverage sequencing strategies [whole-genome re-sequencing (WGS)–P1 and P2; genotyping by sequencing (GBS) P1-P3; Massive Analysis of cDNA Ends transcript sequencing (MACE) P1-P3]. For each of these three methods, the allele frequency was accessed on SNP and haplotype scales. We used three different haplotyping approaches [Gene-based (GH), Marker-based (MH), and Contigs] to estimate and improve a haplotype’s allele frequency in calculated genomic window sizes (HAF, Fig. 2). WGS, MACE, and GBS were compared on all four haplotype scales to illustrate the challenges and trade-offs of each approach. Furthermore, our goal was to validate each *sequencing method* × *haplotype scales allele frequency estimation* combination to an individual genotyping allele frequency (by KASP markers).

### Polymorphism and haplotype yields

The coverage levels and numbers of detected polymorphic loci varied with the genotyping approaches. MACE produced on average 8 million reads across the three pool samples, ranging from 17 to 68 bp length. The reads were trimmed before alignment by removing the ten bases on the head of each read. Additionally, all reads shorter than 40 bp were omitted. We identified 13,079 SNPs (average coverage, 25 reads per SNP) between the two parents. These SNPs were merged into 5919 gene-derived haplotypes (GHs) and 3275 marker-derived haplotypes (MHs). The average values of the SNP counts per haplotype were 2 and 3.5, respectively, yielding read coverages of 42 and 70 for GHs and MHs, respectively. We detected 7.06% (± 0.265 SD) genome-wide wild-type donor allele frequency across the three pool samples (Table 1).

**Table 1:** Descriptive statistics for the three sequencing methods genotyping by sequencing (GBS), MACE transcriptome sequencing (MACE), and whole-genome re-sequencing (WGS)

Stats	Haplotyping level	GBS	MACE	WGS
Number of identified haplotypes	SNP	82,435	13,079	3,991,259
	GH	17,026	5919	34,344
	MH	4702	3275	5946
	Contig	483	460	485
Median read count per haplotype	SNP	6	11	9
	GH	39	21	494
	MH	234	36	2440
	Contig	2645	238	74,134
Average read count per haplotype	SNP	28	25	9.1
	GH	137	42	963
	MH	450	70	5443
	Contig	4837	520	74,855
Variance read count over haplotypes	SNP	3329	348	14.91
	GH	62,413	4,109	2 × 10^6^
	MH	4,30,575	9,482	1.46 × 10^8^
	Contig	2,9*10^7^	4,69,851	1.03 × 10^9^
Genome wide allele frequency	SNP	7.48%	7.06%	6.54%
	GH	7.48%	7.09%	6.56%
	MH	7.43%	7.02%	6.56%
	Contig	7.48%	7.06%	6.54%
Median SNP count per haplotype	GH	3	1	55
	MH	8	2	272
	Contig	142	2	8250
Mean SNP count per haplotype	GH	4	2	106
	MH	14	4	598
	Contig	154	26	8216

Haplotyping levels are: SNP—single nucleotide polymorphism (single data point); GH—gene-based haplotype (origin gene annotation model); MH—marker-based haplotype (origin from 9KiSelect genotyping chip); Contig – Contig haplotypes, in the text referred to as CH, windows of 100 kb size

Values indicate the average across all replicates per sequencing method

GBS produced 20 million paired-end reads across all three pool samples, which only required soft trimming. The removal of duplicate reads eliminated > 90% of reads. Therefore, we omitted this step. With an average coverage of 28 reads per locus, 82,435 SNPs were identified and aggregated into 17,026 GHs and 4,702 MHs. Compared with MACE, the average SNP count per GH doubled (4.2), and those for MH increased fourfold (14.33). The read count of MHs was approximately sixfold higher for the GBS-derived haplotype compared with MACE (Table 1). The average genome-wide allele frequency of the replicates was 7.5% (± 0.45).

WGS produced 400 million high-quality reads for each of both pool samples, which did not require quality adjustments. We identified 3,991,259 SNPs with essentially uniform coverage of 9.1 reads per locus, which clustered to 34,344 GHs and 5946 MHs. Relative to the high number of identified polymorphic loci, the average SNP count per haplotype was 25-to-50-times higher than GBS and MACE (106 GHs, 598 MHs), respectively. The high saturation of haplotypes yielded high haplotype read coverages of 963 and 5443 for GHs and MHs, respectively. The average genome-wide allele frequency measured over the two replicates was 6.5% (± 0.14) and was accordingly 0.5% to 0.9% lower than GBS and MACE, respectively (Table 1). The genome coverage of these polymorphic loci is shown in Additional file 1: Fig. S1. MACE yielded a reduced coverage and expression profile in the pericentromeric region of all chromosomes. A less pronounced, similar pattern was observed for GBS, i.e., lower coverage in the pericentromeric region, while relatively higher in the telomeres. In contrast to MACE and GBS, WGS uniformly tagged all regions (telomere and pericentromere) across all chromosomes.

### Haplotype statistics

**Fig. 3:**
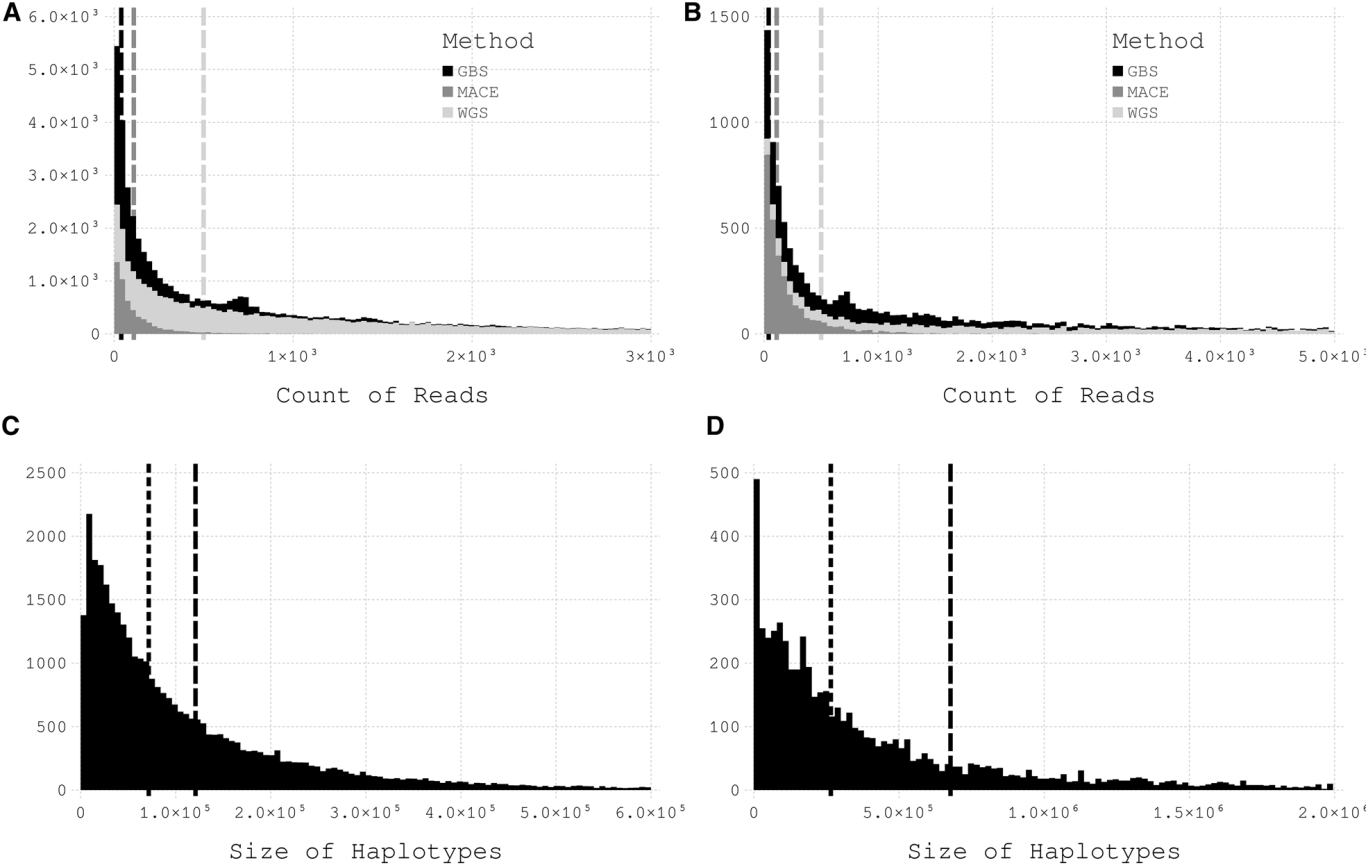
Read count per haplotype for the three applied sequencing methods with their mean value as a vertical line and the distribution of the haplotype window size for gene-based (GH) and marker-based (MH) haplotypes. Mean value across all pool samples. **A** Count of reads per GH for the three applied sequencing methods. **B** same as for (**A**), but based on MH. **C** Size of the GH in base pairs. The dotted line indicates the median value; the dashed line represents the mean value. **D** same as C for MH

On the GH scale, coverages increased to 42, 137, and 963 reads per haplotype for MACE, GBS, and WGS, respectively. Compared with the single SNP coverage level, these values represent 2-, 4-, and 100-fold increases. The mean coverage values of MHs increased to 70, 450, and 5443 reads per haplotype for MACE, GBS, and WGS, respectively (Fig. 3). The haplotype window size is non-static, adjusted according to the distance between markers or annotated genes. Therefore, the dimensions of the haplotypes vary for GHs and MHs (Fig. 3). The mean and median sizes of GHs across all replicates were 216,129 bp and 158,233 bp, respectively, vs. 1,152,370 and 3,944,288 for MHs.

To evaluate the benefits of the haplotyping approach based on genotyping strategies, we asked whether the sizes of constructed haplotypes affect the HAF precision. The extension of haplotypes does not follow a sliding window approach. The haplotype window size is relatively large (illustrated above), raising the question of whether the accuracy of the haplotype frequency depends on the extension size and the chromosomal position. To test this possibility, the deviation of the pool's estimated frequency from the actual frequency was tested using a linear regression analysis compared, where the extension size and chromosomal position were included as cofactors. As a result of this test, the position did not influence the deviation for WGS and MACE haplotypes or the extension size of the WGS and marker MACE haplotypes. For GHs of MACE, a regression with *p* = *0.0065* (regression coefficient = 2.25 × 10^−7^) was determined. MACE only covers expressed 3′ ends of genes and is based on expressed genes. Therefore, the bias may be related to this biological background. For GBS, no regression could be calculated.

### Validation of pool sequencing through single-sample genotyping

One pool (P1) was individually genotyped for 21 loci to validate pool haplotype allele frequencies. KASP markers had a failure rate < 0.015 (19 of 21) and were compared with the corresponding pool of genotypes using the Pearson correlation, root-mean-square-error (RMSE), and a negative binomial zero-inflated linear model (Additional file 1: Table S1). We expected this would assess the authenticities of the identified pool allele and haplotype frequencies.

The Pearson correlation of the MACE-pool SNP allele frequencies compared with the KASP-derived allele frequencies was 0.79, which increased to 0.9 and 0.93 for the GH and MH haplotypes, respectively (Additional file 1: Fig. S2). A similar pattern was observed for the WGS dataset, in which 11 SNPs, 17 MHs, and 15 GHs matched the KASP markers (r^2^ = 0.93, SNP; 0.97, GHs; 0.96, MHs) (Additional file 1: Fig. S3). Consistent with the increased correlation using haplotyping, the RMSE was lower for GHs and MHs in MACE and WGS (Table 2). The same was true for the negative binomial model in which haplotyping compensated for low coverage sequencing. The deviation of the GH set was the smallest compared with the single genotyped reference set, followed by the MH set. On the scale of single SNPs, a significant difference with a probability threshold of 0.05 for the negative binomial model was detected for WGS.

**Table 2:** Individual genotyping of selected KASP markers compared to pool sequencing (P1)

	Haplotyping level	GBS	MACE	WGS
RMSE	SNP level	–	0.07	0.15
	GH level	0.11	0.04	0.03
	MH level	0.06	0.04	0.03
	Contig level	0.03	0.04	0.03
Stat test negative binomial level	SNP level	–	0.67	0.02
	GH level	0.35	0.99	0.57
	MH level	0.47	0.94	0.39
	Contig level	0.59	0.28	0.46
Stat test zero inflated level	SNP level	–	0.68	0.09
	GH level	0.15	0.64	0.64
	MH level	0.62	0.67	0.63
	Contig level	0.44	0.62	0.53
*Pearson* Residual width	SNP level	–	3.85%	3.37%
	GH level	3.78%	3.70%	3.49%
	MH level	3.77%	3.89%	3.40%
	Contig level	3.60%	3.92%	3.95%
*Pearson* correlation	SNP level	–	0.79	0.93
	GH level	0	0.9	0.97
	MH level	0.83	0.93	0.96
	Contig level	0.94	0.88	0.95
Median read coverage of haplotypes in pool sample per KASP marker	SNP level	41	51	7
	GH level	51	82	607
	MH level	575	158	7749
	Contig level	10,122	917	81,186
Count of matched KASP markers to pool seq	SNP level	1	19	11
	GH level	15	16	15
	MH level	17	17	17
	Contig level	19	19	19

Genotyping by sequencing (GBS), MACE transcriptome sequencing (MACE), and whole-genome re-sequencing (WGS) (P1 sample). Haplotyping levels are: SNP—single nucleotide polymorphism (single data point); GH—gene-based haplotype (origin gene annotation model); MH—marker-based haplotype (origin from 9KiSelect genotyping chip); Contig – Contig haplotypes, in the text referred to as CH, windows of 100 kb size. RMSE = root mean square error of pool to individual genotyping on different haplotyping levels for three different genotyping approaches. *Stat test* rows present the *probability* value, where *p* < *0.05* indicates significant variations between individual and pool genotyping. *Pearson* residual width—average deviation of pool haplotype allele frequency estimate to individual genotyping. *Pearson* correlation—correlation of pool to individual genotyping, for each haplotyping level and genotyping approach

**Fig. 4:**
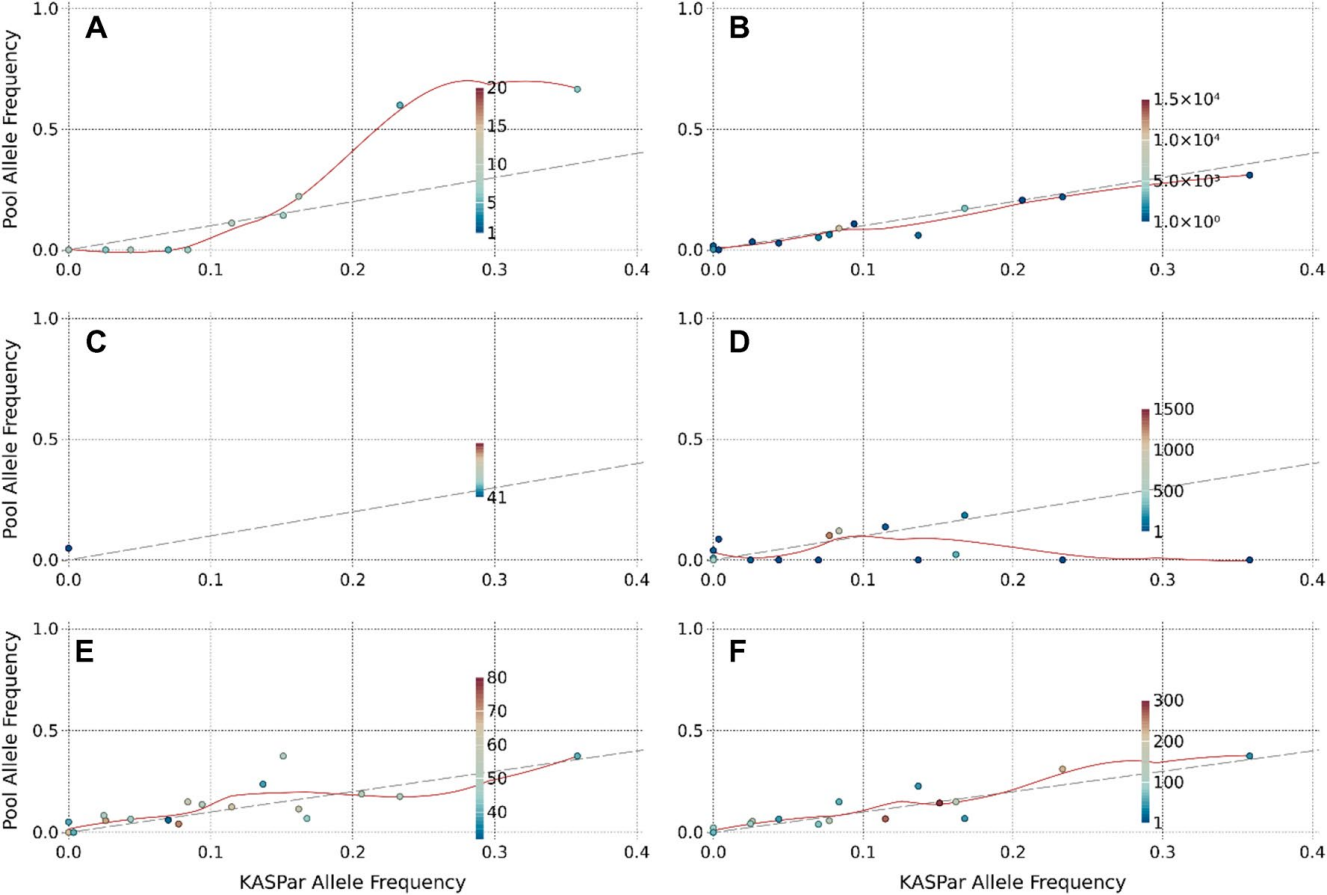
The pool obtained allele frequency (P1) for three tested sequencing strategies (WGrS, GBS, MACE) correlated to individual genotyping (KASP). Three different methods were compared on their accuracy for pool sequencing of large pools. **A** SNP-based pool AF compared to the KASP assay detected allele frequency for WGrS. **B** Gene haplotype-based pool AF compared to KASP assay for WGrS. **C** SNP-based pool AF to KASP for GBS. **D** Gene haplotype-based pool AF compared to KASP assay for GBS. **E** SNP-based pool AF to KASP for MACE RNAseq. **F** Gene haplotype-based pool AF compared to KASP assay for MACE RNAseq. The dotted line indicates the optimal match of the individual (KASP assay) and pool sequencing. Each point represents a single locus**—**for (**A**, **C** and **E**), a locus is a SNP; for B, D and F, a locus is a gene, related to a KASP marker. The colour of the points presents the read coverage of the locus. The red line is a regression through all points

While MACE and WGS showed constant overlaps with KASP results regarding similar allele frequencies, GBS indicated higher deviations. A correlation could not be calculated on the SNP scale because only a single SNP matched the KASP markers. A correlation (− 0.0035) was not observed for GHs, whereas the correlation was 0.834 on the MH scale (Additional file 1: Fig. S4). Generally, RMSE values decreased for all pool sequencing methods by implementing haplotypes (Table 2). The linear model revealed that all haplotyping scales were equal to the KASP markers. This was not true for the SNP scale of the WGS dataset. In summary, the precision achieved by gene-based HAF was superior to the single SNP allele frequency in each genotyping method (Fig. 4).

**Fig. 5:**
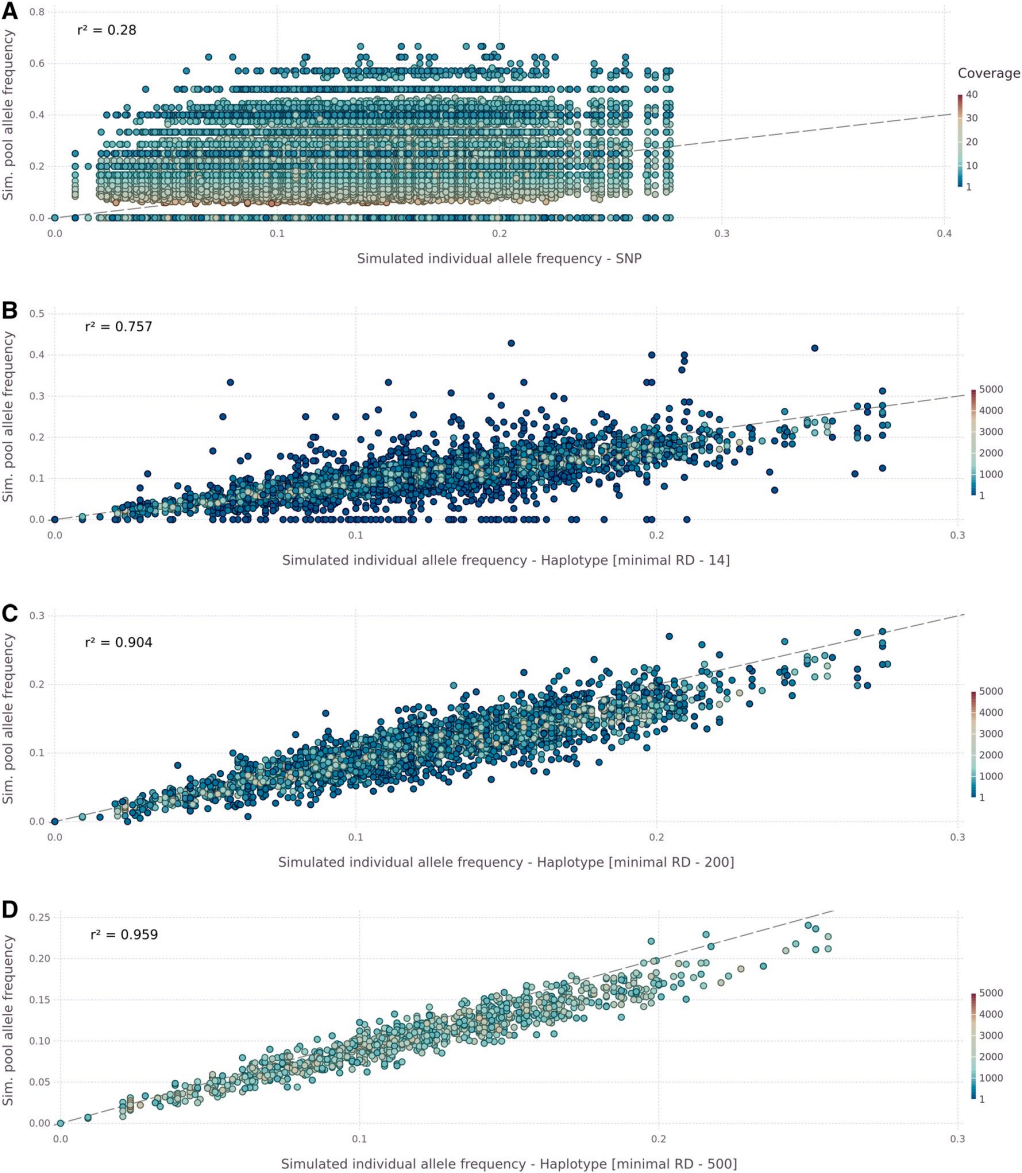
The correlation of simulated individual allele frequency to the simulated pool allele frequency in a simulated BC_2_F_3_ population. The y-axis presents the pooled sample allele frequency on different levels, while the x-axis shows the ‘true’ individual genotyping derived allele frequency. The dashed line indicates the optimal fit, while the color illustrated the read coverage level per SNP/ haplotype. **A** comparison of SNP-based pool sample allele frequency against the individual genotyping allele frequency. **B** Comparing HAF without a minimum threshold for read coverage per haplotype (min reads observed**—***12*). **C** Comparing HAF to the individual genotyping allele frequency. on a minimum coverage level of *200* reads per haplotype. **D** Comparing HAF on a minimum coverage level of *500* reads per haplotype

Although allele frequencies of KASP highly correlate with haplotype-based frequencies, comparing a higher number of loci would be preferred. For this purpose, the allele frequencies of 300 genotypes of a BC_2_F_3_ population were simulated for all markers included in the 9KiSelect chip array [27]. The population was constructed according to markers with a unique physical location (4929 markers). Subsequently, a pool sequencing sample was generated to validate the observations of the experimental populations. The simulated pooled sample was generated on a sequencing depth similar to the WGS approach (10 × coverage per SNP). Additional pseudo SNPs were added to the marker linkage window (MLW) surrounding the chip markers so that the HAF estimation could be carried out analogously to the experimental data sets, applying the model illustrated in Fig. 2. Therefore, we found in MLW 43 SNPs (median), which led to median read coverage of 515 reads per haplotype. Generally, we identified 245,732 SNPs after quality filtering (Qual > 100), including 4,003 SNPs from the 9KiSelect chip. The SNP’s allele frequencies of SNPs located in the same MLW were aggregated to derive a HAF value for each MLW. Additionally, we compared the simulated individual genotyping against the simulated pool genotyping allele frequency. We found a low correlation in the direct comparison of SNPs (r^2^ = 0.28) while haplotyping in the MLW to a HAF value increased the Pearson correlation (Fig. 5). Depending on the minimal read coverage threshold, we observed correlations up to 0.96. We found that haplotypes with > 200 read coverage correlated with > 0.9, while lower read depth indicated decreased correlations. Similarly, the RMSE decreased from 0.12 (SNP) to 0.021 (haplotype, ≥ 500 read coverage). The maximum correlation, accompanied by the lowest RMSE, was found for haplotypes with a minimum coverage of 500 reads (r^2^ = 0.959, RMSE = 0.021).

### Comparisons of contig-based haplotype allele frequency estimations

A simplified contig-based haplotyping approach (CH) [24] was compared with MHs and GHs for relevant deviations of the HAFs. CH yielded 12-to-31-fold higher coverage compared with the MH method. The higher coverage per CH is attributed to a 10-to-83-fold lower count in haplotype objects (485 contig haplotypes vs. up to 35,000 gene haplotypes) (Table 1). The CH allele frequency was compared with the KASP markers, revealing correlations of 0.94, 0.88, and 0.95 for GBS, MACE, and WGS, respectively. The linear model did not show variation between the allele frequencies of KASP and CH, indicating the CH achieved an adequate fit, equivalent to those of MH and GH.

Nevertheless, a marginally higher RMSE of the CH was observed compared with MACE and WGS. Similarly, the correlation to individual genotyping was slightly lower compared with GHs and MHs. A relevant improvement over MHs and GHs was observed only for the GBS pools (Table 2). These findings emphasise that increased coverage and larger haplotyping windows do not necessarily achieve higher precision.

### Reproducibility

According to Antonovics [28], we assumed that 300 genotypes in a pool sufficiently represent the entire population of 25,000 genotypes. Therefore, we hypothesised that biological replicates of the same population will highly correlate if genotyping does not introduce a significant error. This hypothesis generally held true for WGS, in which the correlations between the replicates were 0.91 and 0.96 for GHs and MHs, respectively (Table 3). However, the correlations of replicates in MACE and GBS were 0.64 and 0.59 for GHs and 0.75 and 0.67 on the MH scale. MACE and GBS revealed significant variations between the replicates on the negative binomial scale of the model, where MACE < GBS. The zero-inflated model showed significant variation of the GH scale of WGS but was not significant at the MH scale (p = 0.2) (Table 3). Each pool genotyping method indicated a reduced sample correlation at the single SNP scale, likely associated with limited read coverage.

**Table 3:** Comparison of the replicates for each sequencing method

**Replicate comparison**	Haplotyping level	**GBS**	**MACE**	**WGS**
Pearson correlation	SNP level	0.55	0.5	0.13
	GH level	0.64	0.59	0.91
	MH level	0.75	0.67	0.96
	Contig level	0.75	0.9	0.99
Negative Binomial	SNP level	< 0.001	< 0.001	< 0.001
	GH level	< 0.001	0.007	0.67
	MH level	0.17	0.35	0.52
	Contig level	0.91	0.46	0.35
Zero inflated	SNP level	< 0.001	0.01	< 0.001
	GH level	< 0.001	0.01	< 0.001
	MH level	< 0.001	0.76	0.2
	Contig level	0.48	0.06	0.03

Genotyping by sequencing (GBS), MACE transcriptome sequencing (MACE), and whole-genome re-sequencing (WGS). Haplotyping levels are: SNP—single nucleotide polymorphism (single data point); GH – gene-based haplotype (origin gene annotation model); MH—marker-based haplotype (origin from 9KiSelect genotyping chip); Contig—Contig haplotypes, in the text referred to as CH, windows of 100 kb size. *Pearson* correlation—correlation of the pool genotyping replicates (P1-P3) to each other, for each haplotyping level and genotyping approach. Negative binomial/zero inflated—probability values from a generalized linear model, based on a negative binomial and zero inflated distribution. Both distributions are necessary to cover the entirety of the allele frequency distribution per locus

### Genome-wide allele frequency variations

Pool sequencing can be used to identify the rate of gene flow, random drift, and natural selection. The applied pool genotyping approaches' reliabilities were tested by calculating the average allele frequency distance between two neighbouring haplotypes.

A given BC_2_-derived population of self-pollinating barley depends on the history of BC_2_ families. For example, BC_2_F_1_ possesses approximately 25% heterozygous regions, organised through recombination between distinct blocks during crossing. These heterozygous regions are integrated into the remaining homozygous regions. During subsequent generations, these heterozygous blocks will follow the degradation of heterozygosity through selfing as well as through a putative effect of selection or other forces that alter allele frequency [28]. The linkage blocks corresponding to founder subfamilies should therefore be large. Consequently, the allele frequencies between neighbouring haplotypes are often identical or marginally different, as these haplotypes are smaller than the linkage blocks. Single haplotypes with a deviated allele frequency, compared with their neighbours, are likely false-positive hits and therefore should be disregarded, as a double crossing-over event is doubtful.

Most haplotypes with a high allele frequency deviation compared to their neighbours were observed for GBS (25.97% of haplotypes, median HAF deviation 1.87%), followed by MACE (21.57%, median = 2.4%) and WGS (13.3%, median 1.14%). These results are supported by the observation that GBS (8.7% per haplotype) and MACE (8.9%) yielded the highest mean variation between two haplotypes compared with WGS (Additional file 1: Fig. S5).

**Fig. 6:**
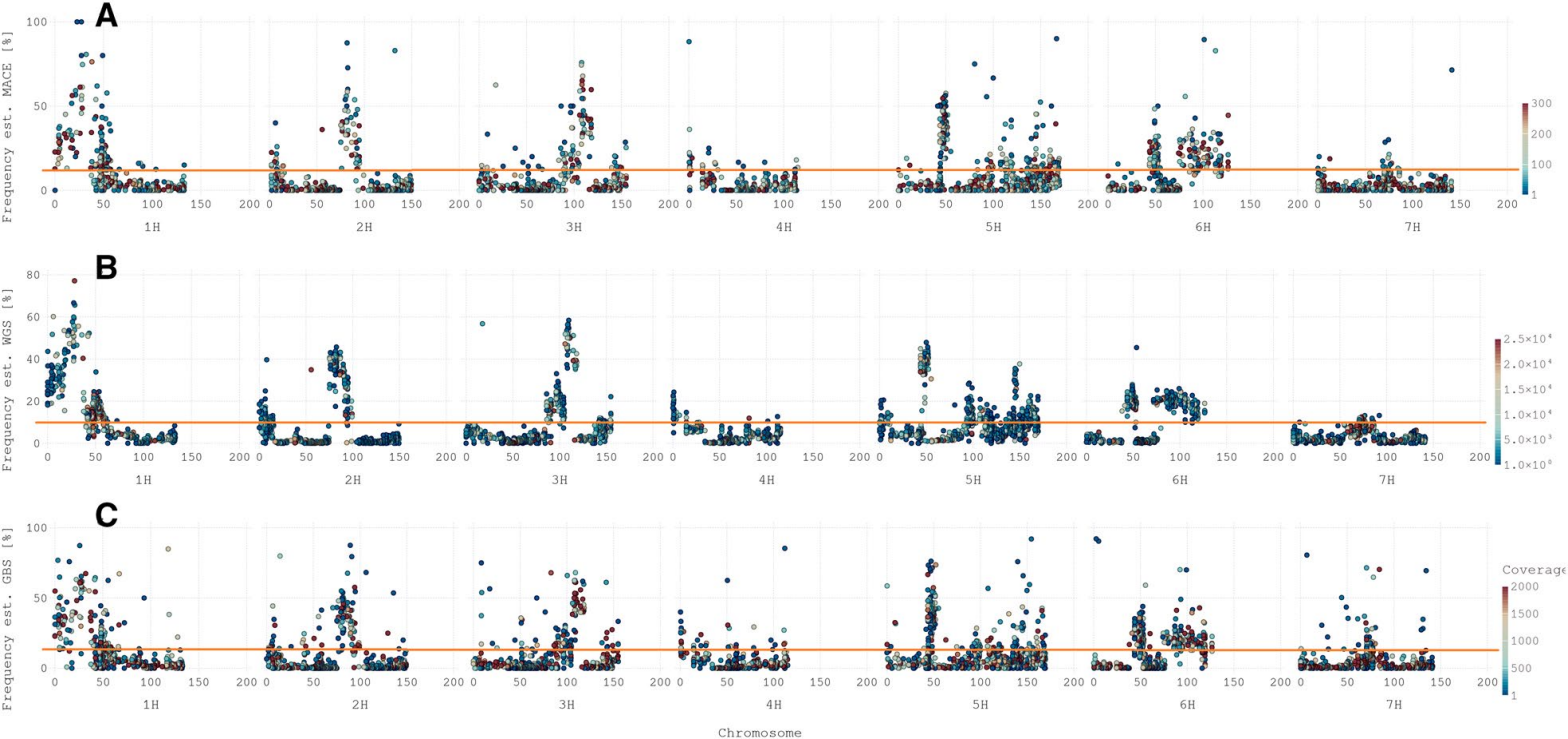
Genome-wide allele frequency on a genetic map for MH. The average donor allele frequency across all replicates (P1-P3) is plotted in % (y-axis) against the genomic position (x-axis), split by chromosome and illustrated in cM. Each dot represents an MH, and the color is related to the read coverage. The orange line indicates the expected allele frequency in the BC_2_F_3_. **A** MACE RNA sequencing output, **B** WGrsS, **C** GBS. Values are the average across all replicates

Mapping the HAF on a genetic (Fig. 6) and a physical map (Additional file 1: Fig. S6) highlight selected regions as well as likely allele frequency call errors. The three genotyping strategies indicate similar success associated with the detection of macro-window allele frequency patterns. Such windows include multiple haplotypes (dozens to hundreds). A HAF deviating from the initial allele frequency of the population (12.5%) (Additional file 1: Fig. S6) was observed for most regions. The above initial donor allele frequencies were observed on the short arms of chromosomes 1H, 4H, and 6H, as well as on the long arms of chromosomes 2H, 3H, 5H, and 6H (Fig. 5).

Further, an increase in the donor HAF was observed in the pericentromeric region of chromosome 5H. Thus, peaks identify selection sweeps with a high fitness advantage at some of these regions. Conversely, the donor allele frequencies of multiple regions decreased to 0 or close to 0. These patterns were observed in all eight sequencing libraries. Nevertheless, the qualities of GBS and MACE decayed, indicating that the most consistent picture was achieved using WGS.

To validate the precision of the three pool genotyping methods, we examined the brittleness locus on chromosome 3H [29]. The donor parent is characterised by the physiological trait of ear brittleness, which is likely negatively selected by combine harvesting. Further, phenotyping of the population revealed no measurable brittleness alleles in the population (BC_2_F_21_). Barley is a self-pollinating crop that should be highly homozygote in the tested generation. Therefore, dominance effects should not mask the donor phenotype. Additional file 1: Fig. S7 shows that the donor allele frequencies of this region determined using WGS, GBS and MACE were 0.01286, 0.0317, and 0.0205, respectively, indicating adverse selection within this region, most likely attributed to the brittleness locus.

## Discussion

Here we compared three pool genotyping methods (GBS, MACE, WGS) for their suitability for application to pooled genotyping. Our immediate goal was to achieve a median sequencing coverage of 10 reads per locus for each pool genotyping strategy. Our overall goal was to precisely estimate the allele frequencies of a population on a gene scale while limiting sequencing depth. This goal was ultimately achieved by a median coverage per genotype in the pooled sample of approximately 0.03 reads per genotype per variant.

Pooled sequencing allows genotyping a larger population size without increased costs associated with sample size. For the population sample size analysed here, using a genotyping chip compared with the pool sequencing strategy would have incurred 30-fold higher genotyping costs (compared with WGS), accompanied by a reduction of detected polymorphic loci by approximately 99%. We sought further cost reductions through the application of targeted sequencing approaches. We found that the GBS and MACE genotyping strategies were 3- and twofold less expensive, respectively. Moreover, we conclude that WGS was by far the most economical approach ($0.18 per 1,000 variants). In contrast, GBS, MACE, and a potential genotyping chip would cost $2.8, $27, and $354 per 1,000 variant loci, respectively. Nevertheless, individual genotyping may provide information beyond the population allele frequency, useful to address additional hypotheses.

Compared with individual genotyping, collecting sample tissues and preparing DNA are more complex and error-prone. The contribution of DNA from each individual in the pool is a crucial factor that must be regarded throughout the entire sampling procedure, including high-precision tissue homogenisation. Nevertheless, the total workload was comparable. The overall costs for cultivation, sampling, and DNA extraction are lower because there is less space occupied in the greenhouse, and fewer chemicals and disposable laboratory supplies are necessary to produce a pooled sample.

WGS, MACE, and GBS pool genotyping were compared with 19 KASP assay-designed SNPs. The latter technique was used to determine individual genotypes to assess the accuracy of the pool allele frequency [21]. The transcriptome and whole-genome re-sequencing approaches highly correlated without significant deviations between the pool and individual genotyping methods. The RMSE and linear model indicated an improvement by applying the haplotyping approach, particularly for WGS. Further, a reduced correlation was associated with MACE and GBS between replicated pools and a significant deviation of the gene-haplotyping scale (p < 0.01) using the negative binomial linear model.

The deviation from transcriptomic data may be explained by variations in the transcription of single genotypes in the pool. Similarly, the inability to remove PCR duplicates unbalanced the accurate estimation of allele frequency using GBS. By applying restriction enzymes, all reads of the same locus started and ended at the identical base, making it impossible to remove duplicates without eliminating > 90% of the sequences. Variant calling is sensitive to duplicate removal [30, 31]. Therefore, the observed deviation likely originated from PCR duplicates. Further, the three methods indicate loss of donor alleles in the population throughout selfing. Starting from an initial expected genome-wide value of 12.5%, the global allele frequencies of the wild type decreased to 7%, 7.5%, and 6.5%, respectively, in the 21^st^ generation tested, depending on the applied sequencing technique. Besides this overall loss, we observed some regions with a positive selection of the wild allele, which indicates a positive fitness effect of these alleles in the agriculture environment. Some of these are characterized as peaks, which underlines that wild barley can add beneficial alleles to modern cultivars to enhance its fitness under variable climatic conditions, like exemplary drought [32].

The variations in the genome-wide donor allele frequencies between methods may be explained by varying genome coverage achieved by each genotyping method. For example, GBS and MACE yielded substantially fewer reads in the pericentromeric regions than WGS. The 0.5%–1% discrepancy in the genome-wide allele frequency may be attributed to reduced recombinational events in the centromere [33], which ultimately results in larger linkage blocks and lower chances for positive wild donor allele selection. Extending the haplotypes from a point or location to a region increased the accuracies of analyses of this inbreeding crop. However, the window size might need to be reduced for outcrossing species, as the mean linkage equilibrium is expected to be lower. Reducing the gene boundary extension or incorporating recombination rates into the extension algorithm [34] may solve these problems.

Moreover, the establishment of contig haplotypes provided equally robust HAF estimates compared with GH and MH. Nevertheless, the precision of contig haplotypes did not outperform GH or MH and was associated with the cost of haplotyping large blocks. Annotation-based haplotyping benefits from a more flexible window-sizing approach, in which the average haplotypes are smaller. This helps directly link functional annotations to haplotypes and prevents errors introduced by recombination within the haplotype. Further, these large area spanning blocks hinder the identification of a causal reason for the variations in allele frequency.

The haplotyping approach presented here was further validated through a simulation, which yielded identical results compared with the biological data. These results emphasise the superior precision of the proposed haplotyping method compared to a restriction to single nucleotides at the given sequencing level. The high single SNP’s allele frequency variation in each haplotype block (Error bars in Additional file 1: Fig. S2–4) supports these observations. A single SNP allele frequency is, randomly, any value between 0 and 1 in a heterogeneous sample with a low coverage level. When we combined the reads of multiple SNPs in the same recombination block, statistical power increased as a function of the number of aggregated SNPs.

In summary, the HAF estimation approach achieved precision benefits when applied to the three pool-genotyping methods. The cost–benefit of MACE, accompanied by the advantage that only SNPs in intergenic regions were expressed, was negated by the lowest level of observed SNPs and a potential bias introduced by the expression level. Similarly, GBS provided a cost-efficient platform, which identified sufficient numbers of variant loci on the genome scale. Unfortunately, these SNPs were usually distributed among intergenic regions, and the enzyme-based design of the method was inappropriate for pooling approaches because duplicates could not be removed. WGS, the costliest approach, delivered the best price ratio per capita according to the observed variants. Further, this approach did not introduce transcriptional bias or duplicate removal complications and identified variants affecting the reading frame of transcripts.

The allele frequency estimation was based on SNP calling, a highly complex task [35, 36]. Not all SNPs identified as such by a variant caller are true polymorphisms. For example, SNP detection is quite challenging, particularly because it invariably generates a fraction of false-positive SNPs [35]. We addressed this problem by only using SNPs included in the Ensemble *Hordeum vulgare* SNP database [37]. After this adjustment, we obtained an overall alternative-base to reference-base ratio in pools of approximately 0.45 for WGS and MACE, which is consistent with the expectation of unbiased SNP calling [38]. The results are supported by a study [31] showing that the applied pipeline for read alignment and variant calling is best suited for the genomes of large crops. Further, the sampling error minimisation was accomplished by following previously proposed sampling strategies [23]. We used an equal amount of leaf tissue per plant to avoid bias introduced by the unbalanced template amounts.

Low coverage pool sequencing of a population with a low donor allele frequency is challenging. Therefore, we proposed a statistical test based on two distributions, in which a linear model was based on zero-inflated and negative binomial distributions. When the overall minor allele frequency of a haplotype is low (< 0.05) and comprises multiple SNPs with low coverage, it is very likely to include a substantial number of SNPs when the minor allele frequency is zero. The zero-inflated model can accommodate such false-negative observations. This distribution is commonly used in human resource studies, which show similarities to the problem encountered in these sequencing data [39] and achieves high sensitivity for haplotypes with low SNP coverage. The negative binomial model accommodates loci with donor allele frequencies > 0 [37].

As a source for variant detection in population genetics, pool sequencing was used to analyse model species with small genomes [9, 11, 34]. The chosen sequencing coverage levels in these studies exceed reasonable levels in terms of costs and computational time for large genomes. The method proposed here serves as a valuable extension to close the gap between analyses of large and small genomes. For example, 5 × coverage may suffice [24]. Generally, sequencing coverage can safely be reduced if high numbers of polymorphisms are detected. These are aggregated to a haplotype, and the higher resolution of variants compensates for further decreased sequencing depth. Haplotypes are constructed by incorporating parental information and annotated genes or markers, thereby allowing the direct calculation and estimation of the haplotype frequency of a target gene. This enables linkage of allele frequency variations with selection causality and increases sequence coverage and reliability.

The proposed method is limited to the core indicators' availability: parental haplotypes, reference genome, and a biparental system. However, the implementation of linked reads may overcome such limitations, thereby minimising the requirement for biparental systems [40, 41]. Thus, the combination of genotype pooling, low coverage linked-read sequencing, and the estimation of haplotype allele frequencies may represent key features for investigating the evolutionary genetics of populations at moderate expenses. Furthermore, the best estimates of HAF are reached when the read coverage exceeds values beyond 200 reads.

Alternative approaches [42–44] estimate haplotypes from ultra-low coverage of individual genotypes and predict missing locus information through imputation. Such methods provide information about an individual haplotype and allele for each genotype, facilitating genomic prediction and marker-assisted selection. The imputation approach and pooled sequencing target a comparable sequencing depth per genotype. The costs of both strategies should therefore be equal, despite library preparation costs. Nevertheless, the barley genome is characterised by a high degree of repetitiveness [45], which challenges generating alignments and detecting variants at such low sequencing levels. The limitation of ultra-low coverage is the difficulty in detecting variants [46], particularly when variants missing from available databases are detected [47], which may lead to false-positive or false-negative calls. Although pooled sequencing operates at a similar sequencing depth, the higher coverage in a single sample will improve the accuracy of these calls, particularly when multiple pooled samples are called in the same run [43, 44]. Further, in contrast to the imputation method, pooled sequencing is sufficient for MAF < 0.05, and remaining heterozygosity does not require phasing.

## Conclusions

Our analyses employing three genotyping approaches provide compelling evidence that whole-genome re-sequencing at low coverage serves as the most powerful tool. All three methods are suitable for detecting large allele frequency variations in large genomic blocks. Still, only whole-genome re-sequencing allowed the robust estimation of large and subtle differences in allele frequency variations in small genomic windows.

Parental haplotypes, a reference genome, and anchors for haplotype construction, e.g., a gene annotation model, are required to retain high-quality haplotype allele frequencies. Compared to single SNP allele frequency estimation, the proposed haplotyping improves reliability by magnitudes. The peaks resulting from selection, evolution, drift, or migration identify relevant alleles and selection sweeps.

## Methods

### Population

The comparison of sequencing strategies and the validation of the proposed pipeline was performed using a spring barley population, generated by a double backcross population. The cultivar Golf was used as the recurrent, and the wild-type ISR 42–8 [*Hordeum vulgare subsp. spontaneum* (K. Koch) Thell] as donor parent. The population was established according to [48]. After two generations of seed multiplication, the population was grown for 18 consecutive generations in a 15 × 9 m plot, using seed material from the previous generation to establish next year's population (Fig. 1A, B). A sowing density of 330 kernels/m^2^ comprised approximately 45,000 genotypes per population per year. The center part of the plot was harvested as bulk to generate the following generation (about 25,000 genotypes). This population size was expected to marginalise random selection effects such as genetic drift. According to the crossing scheme, the expected genome-wide donor allele frequency of the BC_2_F_3_ population was 12.5%. Variations over the 18 generations from this value may be attributed to adaptation to the environment.

### Sample size estimation of pools

The major problem for uncovering the total genetic diversity of a population lies in testing a sufficient sample size. We aimed to achieve a precision level of 0.05 with a narrow confidence interval of 0.99. To calculate the required minimum sample size, we applied Cochran's Formula [49]

$$n_{0} = \frac{{Z^{2} pq}}{{e^{2} }}, where q = 1 - p$$

where *n*_*0*_ is the sample size, *Z* is the z-value in the Z table for a given confidence level, *p* is the preliminary information about the donor parent's initial population-wide allele frequency, and *e* is the desired level of precision. The expected genome-wide donor allele frequency of 12.5% requires pool samples with 291 genotypes per generation and environment. Three pools were created for MACE and GBS and two for WGS.

### Sampling genotypes

One set of 288 genotypes (P1) and two sets of 300 genotypes (P2, P3) were prepared, representing three replicates comprising 888 different genotypes sampled for the population. The first pool contained 12 fewer genotypes (n = 288) because DNA was additionally extracted for each genotype separately (3 × 96 per plate) to validate the pool allele frequency estimation by individual KASP genotyping of selected loci. A hole punch was used for the pool samples to collect leaf material to avoid sampling bias caused by differences in tissue size and genotype differences. After vigorously homogenising the leaf tissue, DNA and RNA were extracted using a Peqlab Plant DNA/RNA Mini Kit. As a reference, the two founders were individually analysed using the three methods, applying the same sequencing depth.

### Genotyping approaches

We compared the performance of three different Illumina sequencing strategies to estimate the allele frequencies in pooled samples. The advantages and disadvantages of the sequencing strategies associated with coverage, costs, and polymorphism yield are illustrated in Table 4. RNA sequencing (MACE; GenXPro GmbH, Frankfurt am Main, Germany) [50], genotyping by sequencing (GBS; LGC Genomics GmbH, Berlin, Germany) [51] and whole-genome re-sequencing (WGS, Novogene Co., Ltd., Beijing, China) [52] approaches were selected as genotyping methods. The GBS approach utilises a combination of restriction enzymes that recognise few or numerous substrate sites to fragment DNA before library preparation to 145 bp paired-end reads. In addition, another nonspecific DNA sequencing approach was applied using WGS to generate 150 bp long paired-end reads.

**Table 4:** Overview of strengths and weaknesses of the applied methods on different levels

	MACE	GBS	WGS
Costs	Med	Low	High
Genes covered	Yes	Partly	Yes
Level	RNA	DNA	DNA
SNP count	Low	Med	High
Coverage	Varying	Med	Low
Genome representation	Biased	Biased	All
Sampling error	High	Low	Low

*MACE* transcriptome sequencing, *GBS* enzyme-based genotyping by sequencing, *WGS* whole-genome resequencing (med = medium)

We compared the transcript sequencing approach (MACE) with DNA sequencing approaches for estimating allele frequencies. MACE RNAseq generated single-end reads of varying lengths and was chosen instead of classic RNAseq because of claims that it delivers higher coverage rates at the expense of fewer loci [50] and is based on the TRUEQuant method that identifies PCR duplicates. We chose these three methods because they vary in cost (GBS < MACE < WGS). Generally, we performed three GBS or two MACE analyses for the price of one WGS. Further, the two parents of the population were sequenced using the three methods to generate a reference haplotype.

### Haplotype construction

First, we constructed the haplotypes of two parents, and this information was incorporated to infer the allele frequency in the pool sequencing of the population (Fig. 2). Second, within 200 kb and smaller windows, we assumed that recombination was infrequent in a self-pollination species such as barley.

Tilk et al. [24] proposed a sliding window approach to generate contigs, which ultimately generates the boundaries of haplotypes. The allele frequency information observed for variants in the sliding window was aggregated into a haplotype frequency. We applied this method as a comparative approach, in which contig windows of 100 kb were defined across the barley genome. Further, two locally anchored techniques were introduced and tested for precision.

The first, gene-based haplotypes (GH), is based on the gene annotations derived from the IPK database [53] for low- and high-confidence genes. These were used as an anchor to generate haplotypes directly linked to a function. The 80,554 low- and high-confidence genes listed [50] were utilised as the potential origins of haplotypes. We used 39,735 high-confidence genes for haplotype construction.

The second, marker-based haplotypes (MH), uses the unique physically annotated markers from the 9KiSelect chip as haplotype anchor. As both the GH and MH have a limited genomic coverage, the sequence termini were extended at their 3′ and 5′ ends to create haplotype blocks (Fig. 2). We included SNPs mapped within a block to calculate an haplotype allele frequency (HAF). The extension algorithm extended the gene by 45% of the size of the intergenic region, assuming the latter was > 1000 bp. For overlapping genes, an extension in the direction of the overlap was not performed, and a gap of 10% of the size of the intergenic region was not annotated to neighbouring genes. For example, gene Q, positions 200–560, and gene W, positions 800–850, were expanded. Ten percent of the 240 bp intergenic region was removed, leaving 216 bp, which was divided by 2 (= 108 bp). Therefore, the new end position of haplotype Q was 560 + 108 = 668, and the new start position of haplotype W was 800 − 108 = 692, thereby closing the gap between these two haplotypes to 24 bp. Thus, a SNP at position 590 was considered part of haplotype Q. Without this extension, the information for this particular SNP would have been lost and therefore not available to improve haplotype frequency. Further, markers in the 9KiSelect SNP chip [54] were mapped against the reference genome to define a haplotype region analogous to the algorithm presented for the gene model (Fig. 2). This analysis mapped 6,080 markers to the reference genome.

All reads within a haplotype were used to calculate the HAF,

$$HAF_{p} = \frac{{\sum rd_{k} *freq_{pk} }}{{\mathop \sum \nolimits_{k} rd}}$$

where *p* represents the allele of a specific parent, and *k* is the SNP for the coverage *rd* and frequency *freq* of the *k*th SNP. Marker information was utilised to plot the donor allele frequency on a genetic map.

We used *Julia* (version 1.3.0) [55] to run the workflow. This included the haplotype construction and the allele as well as haplotype frequency estimation. For the SNPs, a minimum coverage level of one read was set, and the minimum SNP quality score of *samtools mpileup* [56] was required to exceed 30 for each polymorphism. The source code for the haplotype frequency estimation is deposited at https://github.com/mischn-dev/HAFcall.

### Pool accuracy estimation

Twenty-one SNP-specific competitive allele-specific PCR **(**KASP) assays (SGS TraitGenetics, Gartersleben, Germany) were selected and designed according to the MACE results to determine the precision of pool sequencing. The P1 set, comprising 288 genotypes, was sequenced genome-wide as a pool and individually for the 21 KASP markers. The KASP markers were homogeneously distributed across the genome with three markers on each chromosome. Pool sequencing allele frequencies of GBS, MACE, and WGS sequencing for a single SNP as well as gene-based, marker-based, and contig-based haplotype approaches were compared to the KASP markers for the P1 pool. In some cases, we were unable to detect the same KASP SNP by the pool sequencing. Therefore, linked SNPs in the haplotype windows of MH, GH, and CH surrounding the KASP marker were constructed to a HAF and compared to the corresponding KASP marker. A linear model was applied to compare pools and sequencing methods based on a zero-inflated negative binomial distribution. For this purpose, we used the *pscl* package [57] in R. The extension of a negative binomial model by a zero-inflated model was required because many SNPs within a haplotype may randomly fail to produce a read. Further, we calculated the Pearson correlation and the RMSE between individually genotyped and pool-genotyped samples and between replicates.

$$RMSEestimated = \sqrt {\frac{{\sum \left( {\left( {pestimated{|} - ptrue} \right)} \right)^{2} }}{n}}$$

The RMSE illustrates the actual deviation to pool estimated allele frequency values and therefore is utilised to elaborate the pooling strategy's accuracy [24]. To investigate the potential unintended influence of bias on the chromosomal position and the extension size, we applied a linear model, where

$$pestimated - ptrue = lm\left( {extension + position} \right)$$

### Simulation of pool sample to validate haplotype frequency

Additional simulations were performed to exclude the possibility that the observed correlation on shallow coverage level was not artefactual. First, a BC_2_F_3_ population was simulated according to the crossings when the experiment was designed. Second, we used the AlphaSim R package to generate a population of 96,000 individuals with genotypic information for 4929 loci [58]. AlphaSim requires a genetic map as input to estimate recombinational events. Therefore, we used the 9KiSelect chip markers with annotated genetic and physical positions. We randomly selected 300 genotypes to generate a pool sample with 10 × coverage. The read simulation was performed using SubreadR, which required a reference genome, and an additional file indicated the relative sequencing depth of this window, according to the simulated allele frequency of the given haplotype by AlphaSim [59]. Consequently, we created an alternative reference genome with a variant base in each of the 4929 loci and added an alternative base at every 1500th base genome-wide. These additional SNPs will occasionally lie in the MLW and, therefore, should have the same allele frequency as the markers themselves. These additional SNPs improved the MLW’s HAF estimate by aggregating the SNPs allele frequency to one HAF value for each MLW.

Additionally, markers were removed if the haplotype window was < 500 bp or > 1 M bp. We limited the haplotype block size to a maximum of 1 Mb to reduce computational time. Subsequently, 100-bp paired-end reads in the proportion of the simulated allele frequency of the BC_2_F_3_ population for each locus were generated.

Similar to the analysis of the experimental pool samples, the reads were aligned to the barley reference genome, quality-filtered, and variants were called. Variants with a quality higher than 100 were selected to construct single SNP allele frequency and HAF estimates and compare the individual genotyped to the pooled sample. Generally, the HAF of the 300 genotype subsample and the entire population across the entire genome were equal (ANOVA p = 0.89), consistent with a *Pearson* correlation = 0.96.

### Mapping and polymorphism detection

All reads were mapped to the *Hordeum vulgare* reference genome *toplevel v2* [60] using *bwa mem* (version 0.7.1 [61]), and sequence quality was assessed using *fastqc* [62]. Sequences were trimmed as required [63]. Stringent filtering of aligned reads was applied using *sambamba view* (version 0.6.6 [64]), only retaining single mapped reads with a mapping quality > 30 as follows:

*sambamba_v0.6.6 view -h -f bam -p -F "mapping_quality >  = 30 and not (unmapped or secondary_alignment) and not ([XA]! = null or [SA]! = null)"*

Next, duplicates were removed from MACE seq and WGS datasets through *sambamba makrdup* and then sorted using *sort*. Variant calling employed *samtools mplieup* and *bcftools call* (version 1.8 [56]) on minimum variant base quality (Phred quality score = 25). Further, the flags -t AD and –positions were specified. AD, the allelic depth format, generated information about read calls for each allele and sample. The positions flag was used to restrict the pileup to sites where the parents had different homozygote alleles, and the position was reported as a variant locus in the Ensemble variant reference database [65]. Finally, the bcftools options -v and -m were used to generate a vcf file. Only SNPs were called, and indels were omitted from the calls. As previously described [31], the proposed method is the best routine for the variant calling of large plant genomes because of its superior specificity and sensitivity.

## Supplementary Information

**Additional file 1:** **Figure S1.** Distribution and coverage of detected polymorphisms over the barley chromosomes. **A** whole-genome sequencing; **B** MACE transcriptome sequencing; **C** genotyping by sequencing. Values illustrate the average across the replicates (P1**–**P3). **Figure S2.** KASP individual genotyping allele frequency results against the measured allele frequency in P1 pool sample for the MACE transcriptome. The dashed line indicates the optimal match, where pool obtained values match the individual genotyping ideally. The read curve is a regression smooth curve using all points. The color of the points indicates the coverage per locus, which ranges from 1 to several thousand. Error bars highlight the entire margin of single SNP allele frequency that contributed to the overall allele frequency of the haplotype. If no error bar is visible, there is only one SNP contribution information to the haplotype. **A** the single SNP comparison. Only one SNP is detected having the exact same position as the KASP markers. **B** the gene-based haplotype allele frequency compared to the individual genotyping. **C** marker-based haplotype pool allele frequency in comparison to true allele frequency (measured by KASP). **D** contig haplotype-based comparison to the individual genotyping. The pool sequenced sample contains the same 288 genotypes that have been tested individually for the 21 KASP loci. As two KASP markers did not meet the quality threshold, they were omitted from the analysis. **Figure S3.** KASP individual genotyping allele frequency results against the measured allele frequency in P1 pool sample for the GBS data. The dashed line indicates the optimal match, where pool obtained values match the individual genotyping ideally. The red curve is a regression smooth curve using all points. The color of the points indicates the coverage per locus, which ranges from 1 to several thousand. Error bars highlight the entire margin of single SNP allele frequency that contributed to the overall allele frequency of the haplotype. If no error bar is visible, there is only one SNP contribution information to the haplotype. **A** The single SNP comparison. Only one SNP was detected having the exact same position as the KASP markers. **B** The gene-based haplotype allele frequency compared to the individual genotyping. **C** Marker-based allele frequency. **D** Contig haplotype-based comparison to the individual genotyping. The pool sequenced sample contains the exact same 288 genotypes that have been tested individually for the 21 KASP loci. As two KASP marker did not meet the quality threshold, they were omitted from the analysis. **Figure S4.** KASP individual genotyping allele frequency results against the measured allele frequency in P1 pool sample for the WGS data. The dashed line indicates the optimal match, where pool obtained values match the individual genotyping ideally. The read curve is a regression smooth curve using all points. The color of the points indicates the coverage per locus, which ranges from 1 to several thousand. Error bars highlight the entire margin of single SNP allele frequencies that contributed to the overall allele frequency of the haplotype. If no error bar is visible, there is only one SNP contribution information to the haplotype. **A** The single SNP comparison. 10 SNP are detected to have the exact same position as the KASP markers. **B** The gene-based haplotype allele frequency compared to the individual genotyping. **C** Marker-based haplotype pool allele frequency comparison to true allele frequency. **D** Contig haplotype-based comparison to the individual genotyping. The pool sequenced sample contains the exact same 288 genotypes that have been tested individually for the 21 KASP loci. As two KASP marker did not meet the quality threshold, they were omitted from the analysis. **Figure S5.** Median haplotype allele frequency (HAF) difference of two neighbouring haplotypes (blue) and the share of haplotypes being highly different to their physical neighbours (> 5 times median, yellow). All tested pool genotyping approaches are illustrated with their replicates. **Figure S6** Genome-wide allele frequency on a physical map for gene-based haplotypes. The donor allele frequency is plotted in % (y-axis) against the genomic position (x-axis), split by chromosome and illustrated in base pairs. Each dot represents a gene haplotype and the color is related to the read coverage. The orange line indicates the expected allele frequency in the BC_2_F_1_. **A** MACE RNA sequencing output, **B** WGreS, **C** GBS. Values are the average across all replicates. **Figure S7.** Donor allele frequency in the region of *brt1* and *brt2* brittleness genes. The donor allele frequency is illustrated for each gene-based haplotype. The color illustrate the coverage per haplotype, while the shape separated the pool genotyping approaches. For each pool genotyping approach, all pools sequenced are illustrated. Phenotypic data of the population indicates unmeasurable levels of brittleness alleles in the population. All three pool genotyping approaches highlight similar observations on haplotype allele frequency levels

**Additional file 2.** **Table S2** Information on the 21 CASP markers used for validation of pool sequencing. Column 'Marker' gives the unique naming of the CASP; Allel e 1 & Allel 2 denote how many of the 288 homozygote genotypes were observed to carry the respective allele. ('Allel 1' count refers to column 'Ref', while 'Allel 2' refers to 'Alt'). 'failed' indicated for how many genotypes the allele identification failed. 'heterozygot' presents the number of genotypes being heterozygote. The 'ratio' illustrated the allele frequency of the ISR42-8 alleles for each CASP marker. 'Chr' and 'Pos' describe the physical position of the CASP markers on the barley reference genome. 'Quality' denotes the observed SNP calling quality from MACE RNAseq sequencing

## Data Availability

Raw variant calling data and processed data can be found under 10.5281/zenodo.4304046Code used to perform the analysis can be found at https://github.com/mischn-dev/HAFcall

## Abbreviations

CH: Contig-based haplotyping
GBS: Genotyping by sequencing
GH: Gene-derived haplotypes
HAF: Haplotype allele frequency
MH: Marker-derived haplotypes
NGS: Next-generation sequencing
RMSE: Root-mean-square-error
SNP: Single nucleotide polymorphisms
WGS: Whole-genome resequencing
MACE: Massive Analysis of cDNA Ends
KASP: Competitive allele-specific PCR
MLW: Marker linkage window
