# Supplementary material for: High-throughput estimation of allele frequencies using combined pooled-population sequencing and haplotype-based data processing
Source: Plant Methods. 2022 Mar 21;18:34. doi: 10.1186/s13007-022-00852-8 (PMC8935755; doi:10.1186/s13007-022-00852-8)
Supplement: Supplementary file 1 — Additional file 1: Figure S1. Distribution and coverage of detected polymorphisms over the barley chromosomes. A whole-genome sequencing; B MACE transcriptome sequencing; C genotyping by sequencing. Values illustrate the average across the replicates (P1–P3). Figure S2. KASP individual genotyping allele frequency results against the measured allele frequency in P1 pool sample for the MACE transcriptome. The dashed line indicates the optimal match, where pool obtained values match the individual genotyping ideally. The read curve is a regression smooth curve using all points. The color of the points indicates the coverage per locus, which ranges from 1 to several thousand. Error bars highlight the entire margin of single SNP allele frequency that contributed to the overall allele frequency of the haplotype. If no error bar is visible, there is only one SNP contribution information to the haplotype. A the single SNP comparison. Only one SNP is detected having the exact same position as the KASP markers. B the gene-based haplotype allele frequency compared to the individual genotyping. C marker-based haplotype pool allele frequency in comparison to true allele frequency (measured by KASP). D contig haplotype-based comparison to the individual genotyping. The pool sequenced sample contains the same 288 genotypes that have been tested individually for the 21 KASP loci. As two KASP markers did not meet the quality threshold, they were omitted from the analysis. Figure S3. KASP individual genotyping allele frequency results against the measured allele frequency in P1 pool sample for the GBS data. The dashed line indicates the optimal match, where pool obtained values match the individual genotyping ideally. The red curve is a regression smooth curve using all points. The color of the points indicates the coverage per locus, which ranges from 1 to several thousand. Error bars highlight the entire margin of single SNP allele frequency that contributed to the overall allele f [file 13007_2022_852_MOESM1_ESM.docx]

# Supplementary Figures


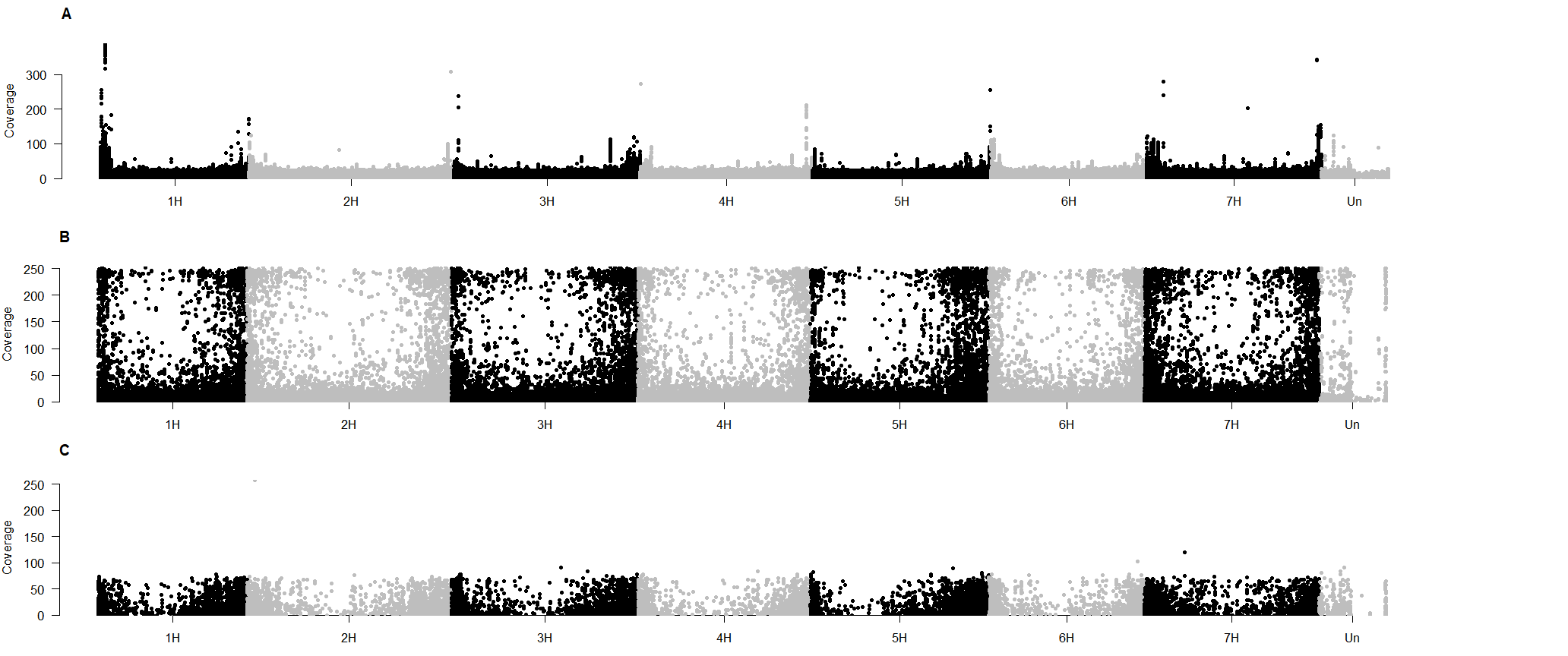


**Supplementary Figure 1 - Distribution and coverage of detected polymorphisms over the barley chromosomes. A**– whole-genome sequencing; **B** – MACE transcriptome sequencing; **C** – Genotyping by sequencing. Values illustrate the average across the replicates (P1-P3).


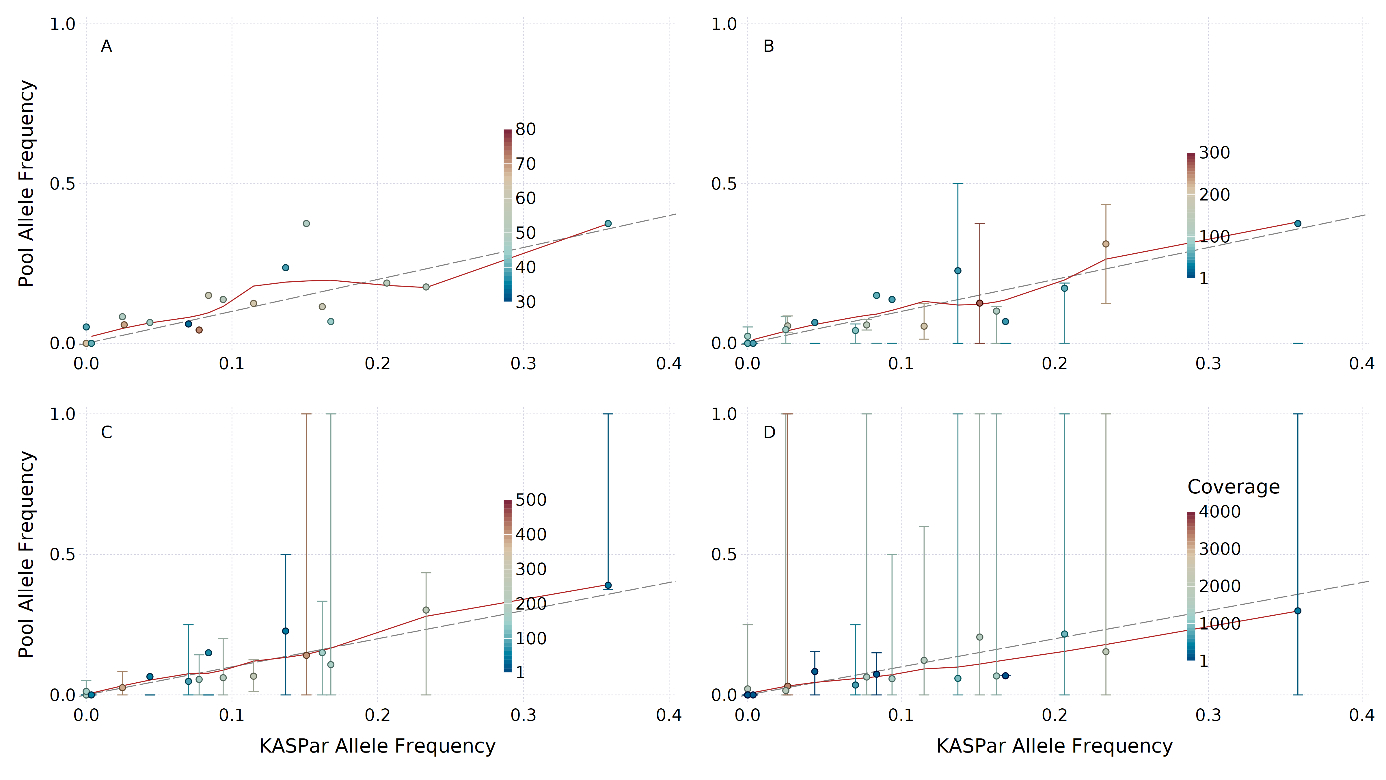


**Supplementary Figure 2 - KASPar individual genotyping allele frequency results against the measured allele frequency in P1 pool sample for the MACE transcriptome.** The dashed line indicates the optimal match, where pool obtained values match the individual genotyping ideally. The read curve is a regression smooth curve using all points. The color of the points indicates the coverage per locus, which ranges from 1 to several thousand. Error bars highlight the entire margin of single SNP allele frequency that contributed to the overall allele frequency of the haplotype. If no error bar is visible, there is only one SNP contribution information to the haplotype. **A –** the single SNP comparison. Only one SNP is detected having the exact same position as the KASP markers. **B –** the gene-based haplotype allele frequency compared to the individual genotyping. **C –** marker-based haplotype pool allele frequency in comparison to true allele frequency (measured by KASP). **D –** contig haplotype-based comparison to the individual genotyping. The pool sequenced sample contains the same 288 genotypes that have been tested individually for the 21 KASP loci. As two KASP markers did not meet the quality threshold, they were omitted from the analysis.


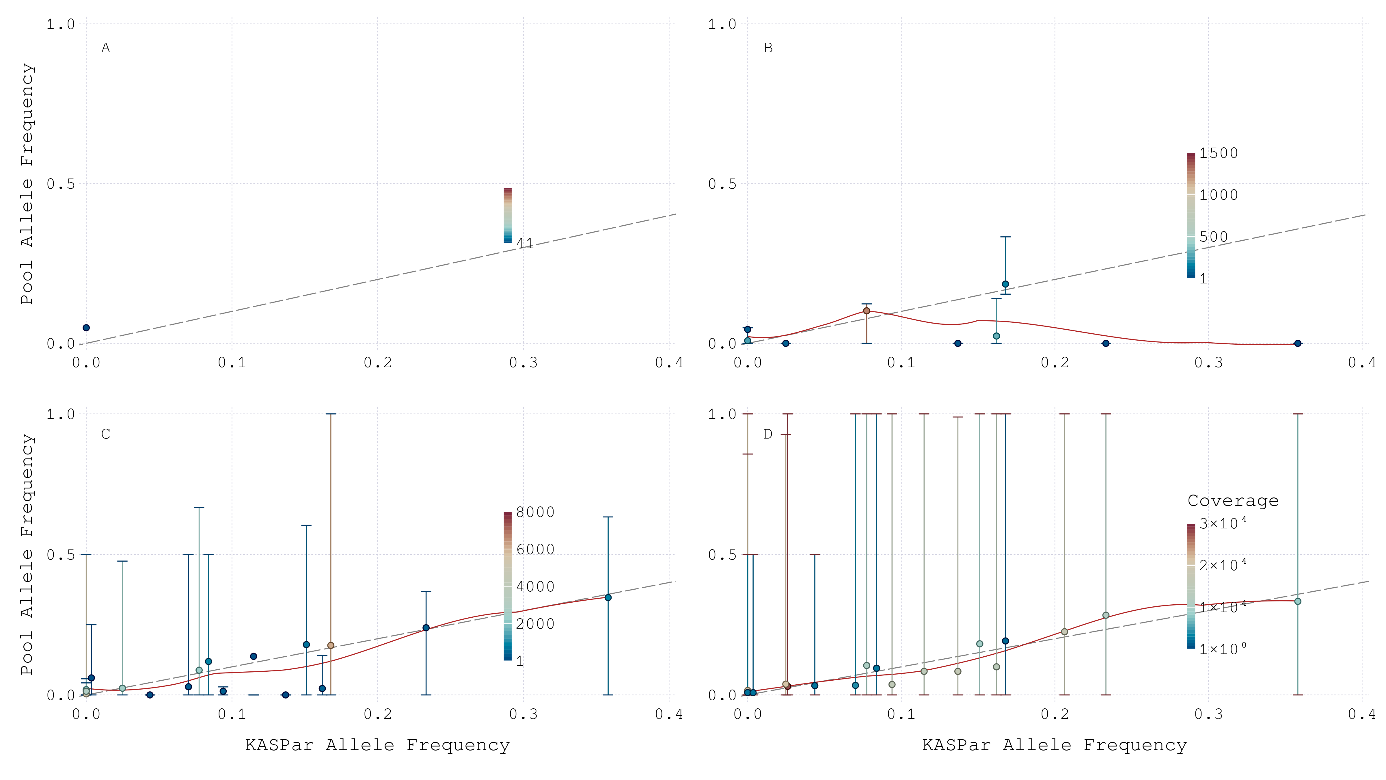


**Supplementary Figure 3 -** **KASP individual genotyping allele frequency results against the measured allele frequency in P1 pool sample for the GBS data.** The dashed line indicates the optimal match, where pool obtained values match the individual genotyping ideally. The red curve is a regression smooth curve using all points. The color of the points indicates the coverage per locus, which ranges from 1 to several thousand. Error bars highlight the entire margin of single SNP allele frequency that contributed to the overall allele frequency of the haplotype. If no error bar is visible, there is only one SNP contribution information to the haplotype. **A –** the single SNP comparison. Only one SNP was detected having the exact same position as the KASP markers. **B –** the gene-based haplotype allele frequency compared to the individual genotyping. **C –** marker-based allele frequency. **D –** contig haplotype-based comparison to the individual genotyping. The pool sequenced sample contains the exact same 288 genotypes that have been tested individually for the 21 KASP loci. As two KASP marker did not meet the quality threshold, they were omitted from the analysis.


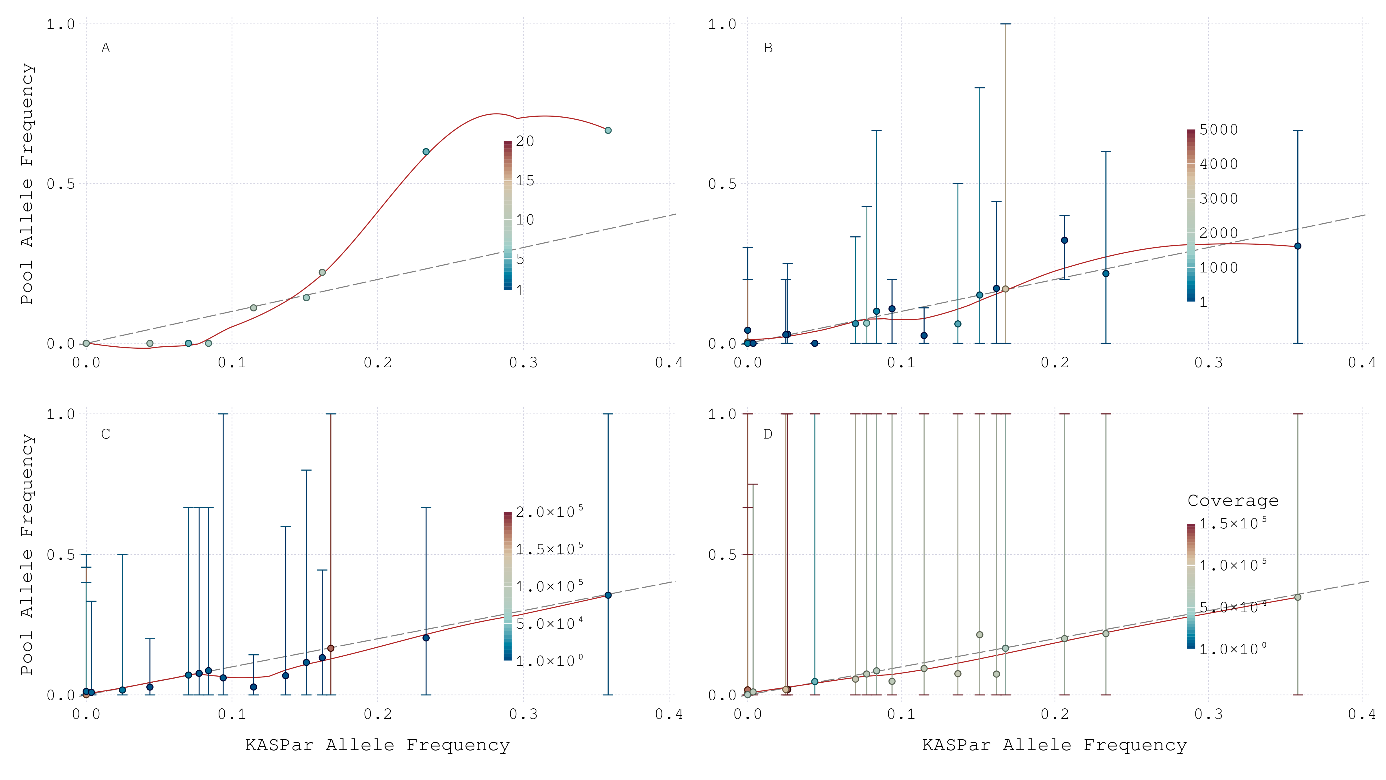


**Supplementary Figure 4 - KASP individual genotyping allele frequency results against the measured allele frequency in P1 pool sample for the WGS data**. The dashed line indicates the optimal match, where pool obtained values match the individual genotyping ideally. The read curve is a regression smooth curve using all points. The color of the points indicates the coverage per locus, which ranges from 1 to several thousand. Error bars highlight the entire margin of single SNP allele frequencies that contributed to the overall allele frequency of the haplotype. If no error bar is visible, there is only one SNP contribution information to the haplotype. **A –** the single SNP comparison. 10 SNP are detected to have the exact same position as the KASP markers. **B –** the gene-based haplotype allele frequency compared to the individual genotyping. **C –** marker-based haplotype pool allele frequency comparison to true allele frequency. **D –** contig haplotype-based comparison to the individual genotyping. The pool sequenced sample contains the exact same 288 genotypes that have been tested individually for the 21 KASP loci. As two KASP markers did not meet the quality threshold, they were omitted from the analysis.


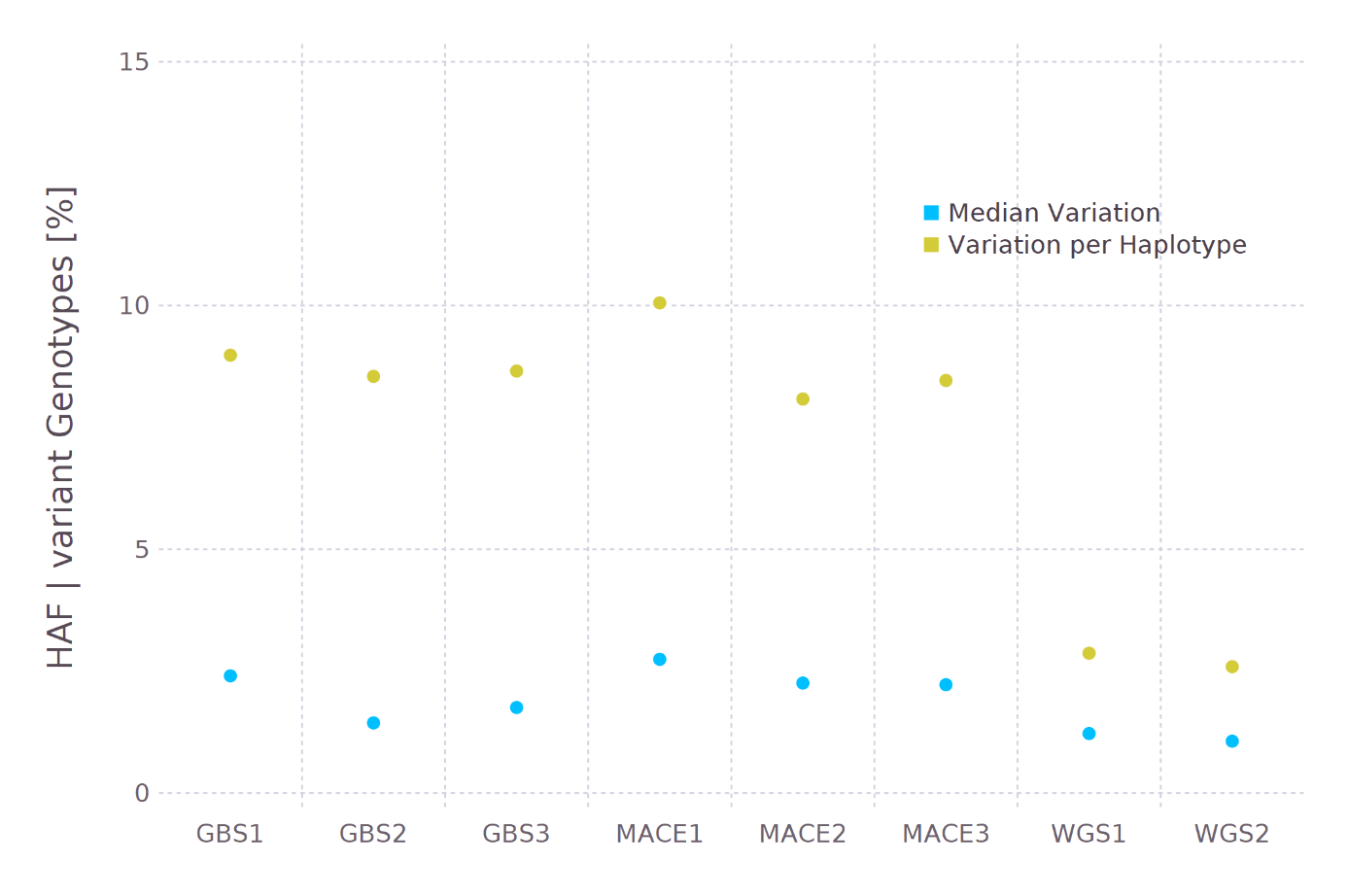


**Supplementary Figure 5:**Median haplotype allele frequency (HAF) difference of two neighbouring haplotypes (blue) and the share of haplotypes being highly different to their physical neighbours (>5 times median, yellow). All tested pool genotyping approaches are illustrated with their replicates.


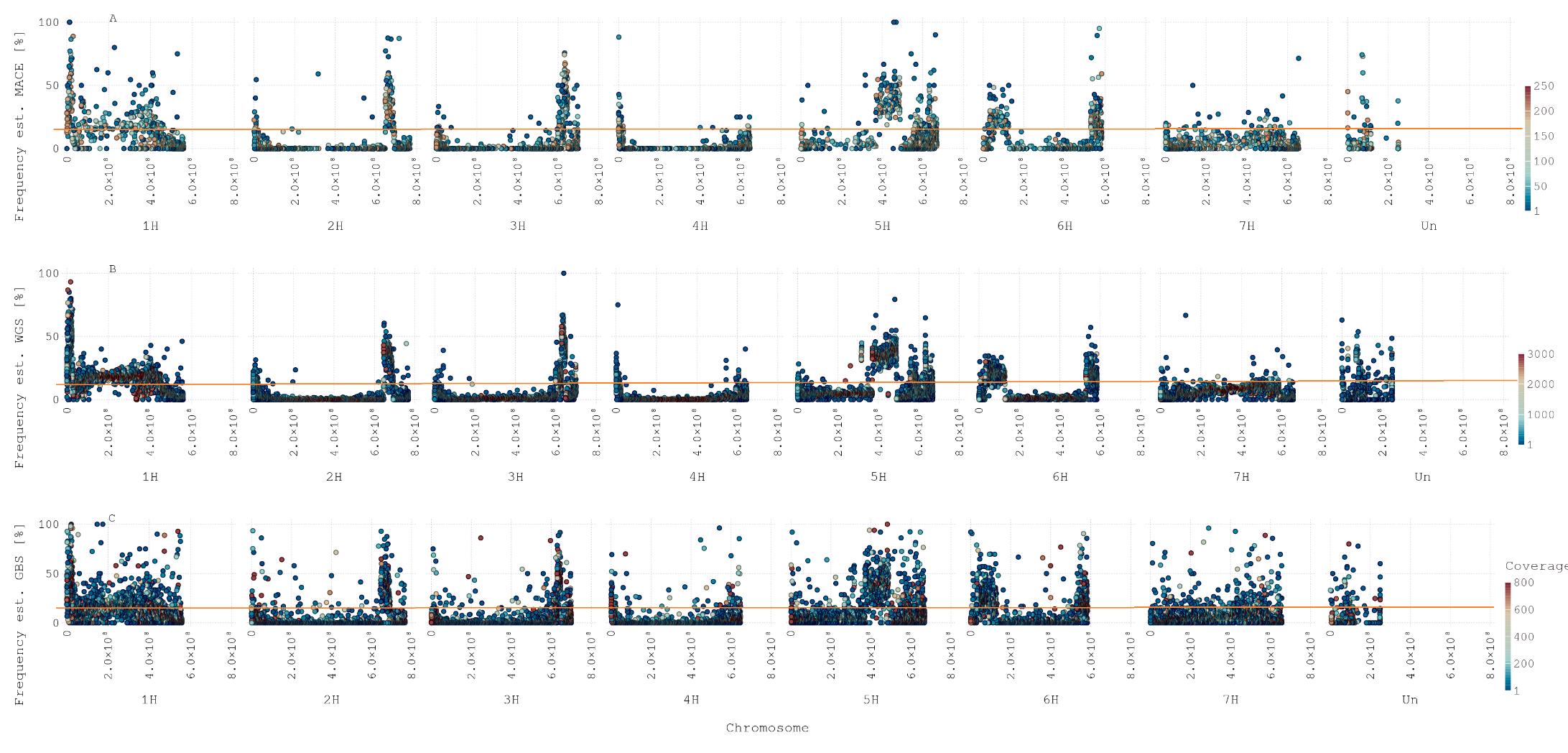


**Supplementary Figure 6 - Genome wide allele frequency on a physical map for gene-based haplotypes.** The donor allele frequency is plotted in % (y-axis) against the genomic position (x-axis), split by chromosome and illustrated in base pairs. Each dot represents a gene haplotype and the color is related to the read coverage. The orange line indicates the expected allele frequency in the BC_2_F_1_. **A** – MACE RNA sequencing output, **B** – WGreS, **C** – GBS. Values are the average across all replicates.


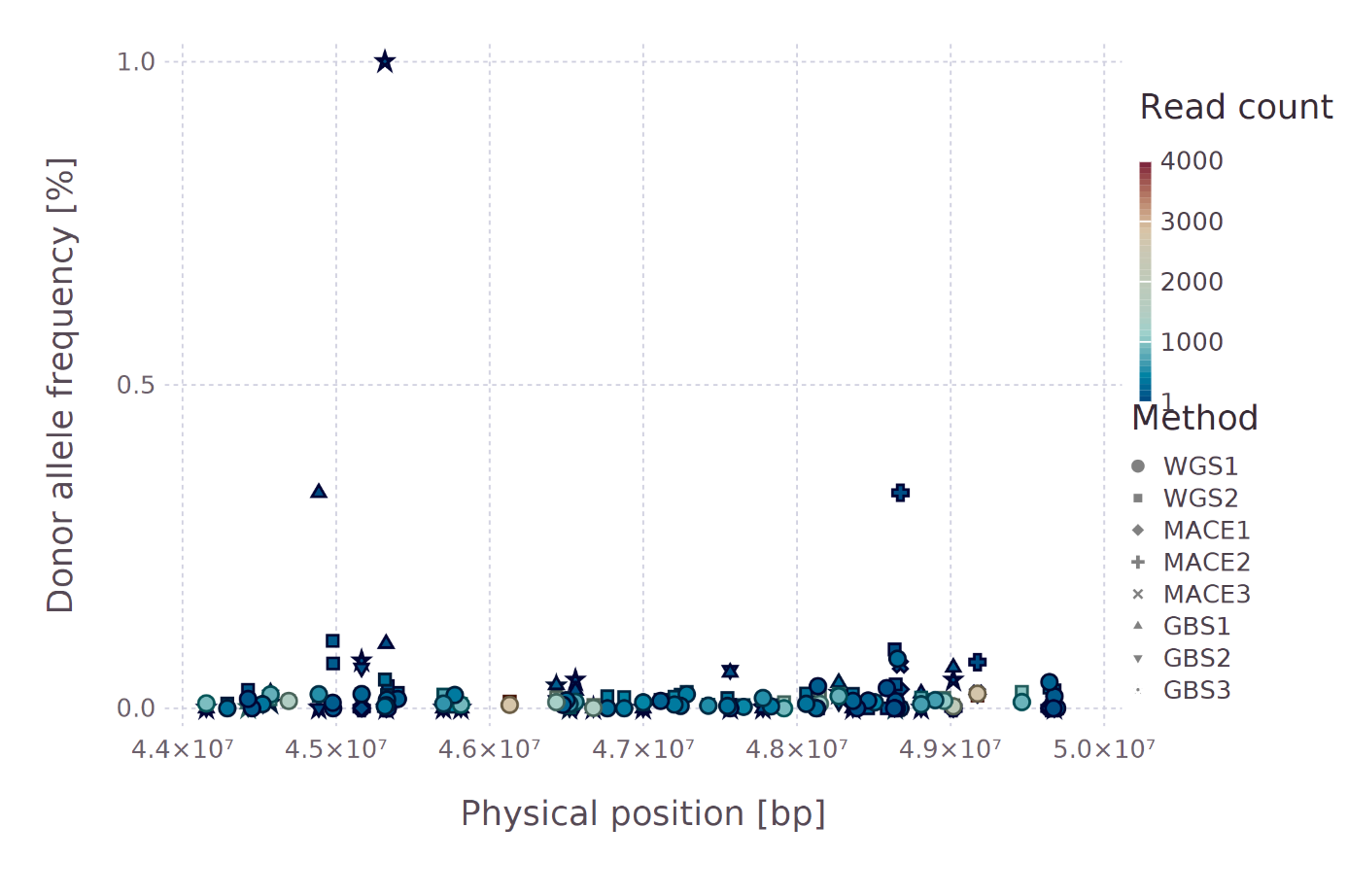


**Supplementary Figure 7: Donor allele frequency in the region of *brt1* and *brt2* brittleness genes.** The donor allele frequency is illustrated for each gene-based haplotype. The color illustrated the coverage per haplotype, while the shape separated the pool genotyping approaches. For each pool genotyping approach, all pools sequenced are illustrated. Phenotypic data of the population indicates unmeasurable levels of brittleness alleles in the population. All three pool genotyping approaches highlight similar observations on haplotype allele frequency levels.
