# Supplementary material for: High-throughput estimation of allele frequencies using combined pooled-population sequencing and haplotype-based data processing
Source: Plant Methods. 2022 Mar 21;18:34. doi: 10.1186/s13007-022-00852-8 (PMC8935755; doi:10.1186/s13007-022-00852-8)
Supplement: Supplementary file 2 — Additional file 2. Table S2 Information on the 21 CASP markers used for validation of pool sequencing. Column 'Marker' gives the unique naming of the CASP; Allel e 1 & Allel 2 denote how many of the 288 homozygote genotypes were observed to carry the respective allele. ('Allel 1' count refers to column 'Ref', while 'Allel 2' refers to 'Alt'). 'failed' indicated for how many genotypes the allele identification failed. 'heterozygot' presents the number of genotypes being heterozygote. The 'ratio' illustrated the allele frequency of the ISR42-8 alleles for each CASP marker. 'Chr' and 'Pos' describe the physical position of the CASP markers on the barley reference genome. 'Quality' denotes the observed SNP calling quality from MACE RNAseq sequencing [file 13007_2022_852_MOESM2_ESM.docx]

Table S1

| Marker | Allel1 | Allel2 | failed | heterozygot | ratio | Chr | Pos | Ref | Alt | Quality |
| --- | --- | --- | --- | --- | --- | --- | --- | --- | --- | --- |
| A_1 | 140 | 37 | 111 | 0 | 0.20904 | chr1H | 9123006 | G | A | 631 |
| A_2 | 45 | 233 | 5 | 5 | 0.167845 | chr1H | 2.5E+08 | G | A | 185 |
| A_3 | 5 | 273 | 6 | 4 | 0.024823 | chr1H | 5.34E+08 | T | C | 208 |
| A_4 | 39 | 246 | 3 | 0 | 0.136842 | chr2H | 11270826 | A | C | 664 |
| A_5 | 0 | 0 | 288 | 0 | NA | chr2H | 42074896 | A | G | 208 |
| A_6 | 98 | 178 | 6 | 6 | 0.358156 | chr2H | 6.74E+08 | T | C | 302 |
| A_7 | 254 | 25 | 6 | 3 | 0.093972 | chr3H | 13621339 | T | G | 665 |
| A_8 | 283 | 1 | 4 | 0 | 0.003521 | chr3H | 3.67E+08 | C | T | 201 |
| A_9 | 42 | 236 | 10 | 0 | 0.151079 | chr3H | 6.28E+08 | C | T | 611 |
| A_10 | 261 | 21 | 4 | 2 | 0.077465 | chr4H | 13536444 | C | T | 208 |
| A_11 | 283 | 0 | 5 | 0 | 0 | chr4H | 3.17E+08 | G | A | 208 |
| A_12 | 284 | 0 | 4 | 0 | 0 | chr4H | 6.3E+08 | A | G | 821 |
| A_13 | 237 | 45 | 4 | 2 | 0.161972 | chr5H | 5799726 | T | C | 847 |
| A_14 | 12 | 273 | 2 | 1 | 0.043706 | chr5H | 3.37E+08 | C | T | 855 |
| A_15 | 250 | 32 | 5 | 1 | 0.114841 | chr5H | 6.43E+08 | G | A | 932 |
| A_16 | 63 | 214 | 5 | 6 | 0.233216 | chr6H | 34026637 | T | A | 203 |
| A_17 | 283 | 0 | 5 | 0 | 0 | chr6H | 3.05E+08 | A | G | 155 |
| A_18 | 224 | 56 | 2 | 6 | 0.206294 | chr6H | 5.6E+08 | A | T | 183 |
| A_19 | 19 | 264 | 3 | 2 | 0.070175 | chr7H | 1.02E+08 | T | G | 176 |
| A_20 | 261 | 23 | 2 | 2 | 0.083916 | chr7H | 3.54E+08 | T | C | 873 |
| A_21 | 275 | 2 | 0 | 11 | 0.026042 | chr7H | 6.42E+08 | C | T | 208 |
